# 2-Aminooxazoles and 2-aminothiazoles: green regioselective synthesis and bioactivity

**DOI:** 10.1039/d6ra07868k

**Published:** 2026-10-19

**Authors:** Pankaj Popatrao Yenare, Rohini Dattatraya Patare, Kailas Khandu Sanap

**Affiliations:** a Department of Engineering Science and Humanities, Marathwada Mitra Mandal's College of Engineering Karvenagar Pune Maharashtra 411052 India pankajyenare@gmail.com; b Radhabai Kale Mahila Mahavidyalaya Ahmednagar Maharashtra India rohinipatare17@gmail.com; c Department of Chemistry, Gokhale Education Society, HPT Arts and RYK Science College Dist. Nashik Nashik Maharashtra 422005 India ksanap100@gmail.com +(0253) 2573097 +912532572153

## Abstract

2-Aminooxazole and 2-aminothiazole are heterocyclic compounds that can be developed to be therapeutically significant in medicinal chemistry as they are associated with a myriad of biologically important multicentric activities such as anticancer, anti-viral, anti-bacterial, anti-fungal, and enzyme inhibition. Chemically, they have been reported to target critical enzymes such as CDKs, aurora kinases, DHPS, and proteases. However, traditional routes to construct heterocyclic compounds are fraught with difficulties, such as poor regioselective control, producing C-4/C-5 regioisomeric mixtures, poor atom economy, laborious purifications, and a substantial waste of solvents. Recently developed green synthetic approaches, such as microwave-assisted synthesis, ultrasound activation, multicomponent reactions, nanocatalysis, and newer techniques that employ syntheses in aqueous media and in a solvent-free manner, have attempted to address these issues. These methods provide better regioselectivity, better reaction efficiencies, and better yields. In the context of synthetic medicinal chemistry, the green approaches enhance the safety and convenience of laboratory operations by decreasing the need for hazardous solvents. However, the absence of the *E*-factor, PMI, and atom economy, as well as the substrate-dependent regioselective variability in these methodologies, has restricted the comparison of these methodologies as a whole, and their application in the pharmaceutical industry on a large scale.

## Abstract 2

This review explores green and regioselective synthesis of 2-aminooxazoles and 2-aminothiazoles, focusing on C4/C5 regioisomerism, synthetic efficiency, biological activities, and future sustainability challenges.

## Graphical abstract

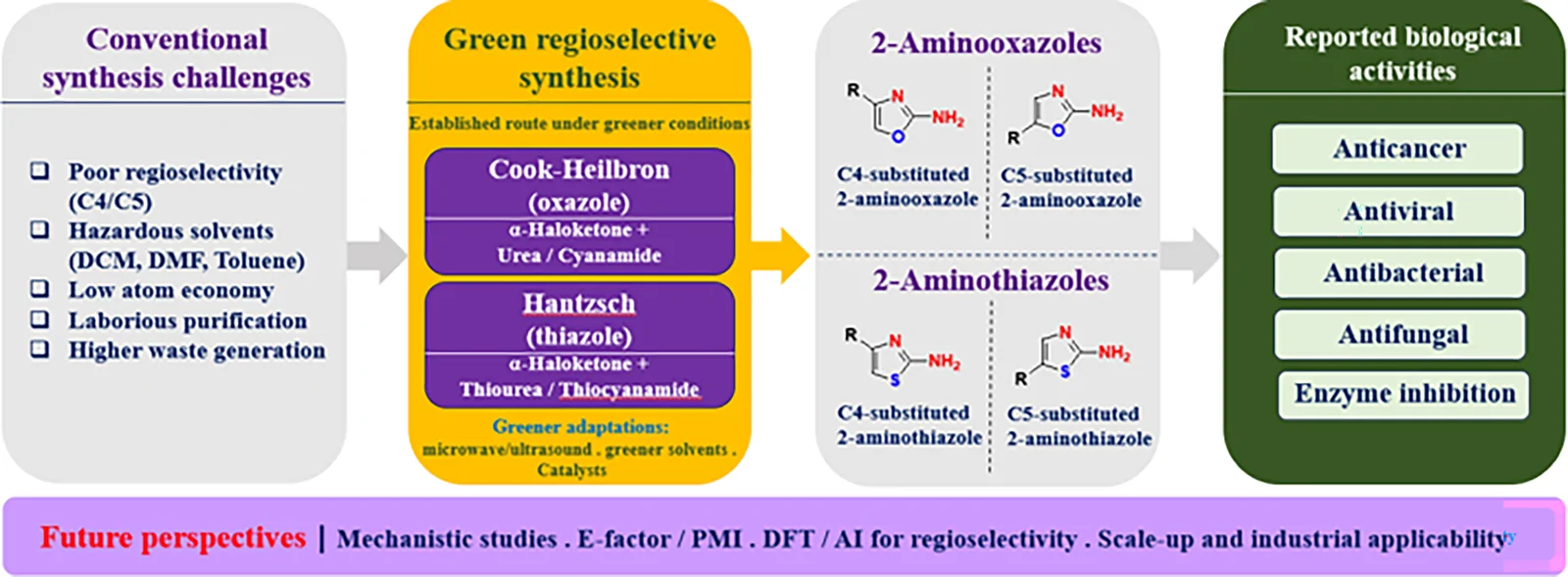

## 1 Introduction

Heterocyclic compounds are widely used in organic and medicinal chemistry, and oxazole (1) and thiazole (2) are among the important five-membered heterocycles in this area. Both rings are found in natural products and in many biologically active molecules, with oxazole containing oxygen and thiazole containing sulfur alongside nitrogen. These rings can also be modified at different positions, making it possible to alter polarity, hydrogen-bonding behaviour, lipophilicity, and electronic properties without greatly increasing the size of the molecule. For these reasons, oxazole and thiazole have remained useful structural motifs in medicinal chemistry and drug discovery.^1,2^
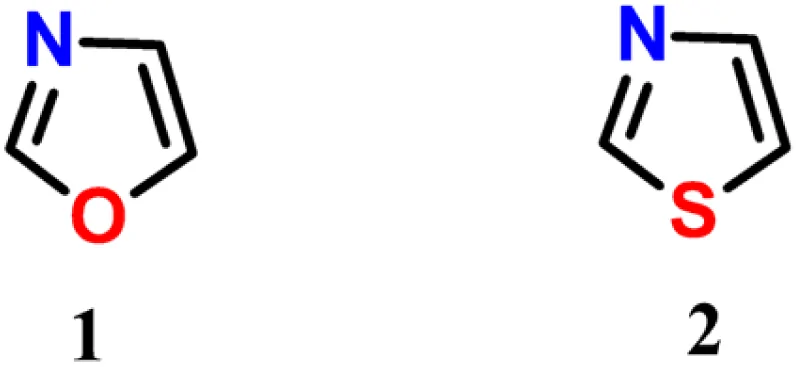


Thiazole occurs naturally in thiamine (3, vitamin B1), in which the thiazolium moiety plays an essential role in the function of the cofactor. Thiazole-containing structures are also found in peptide-derived and marine natural products and have been associated with different biological activities. The relevance of this ring is also evident in medicinal chemistry, as 2-aminothiazole units are present in drugs such as famotidine (4), cefdinir (5), and meloxicam (6) [Fig. 1].^3,4^ Oxazole is likewise found in natural products and biologically active compounds. Oxazole-containing molecules have been explored in several areas of medicinal chemistry, and the ring is present in clinically relevant compounds such as oxaprozin (7). This also demonstrates that the small oxazole ring can be incorporated into structures with useful pharmacological properties.^5^

**Fig. 1:**
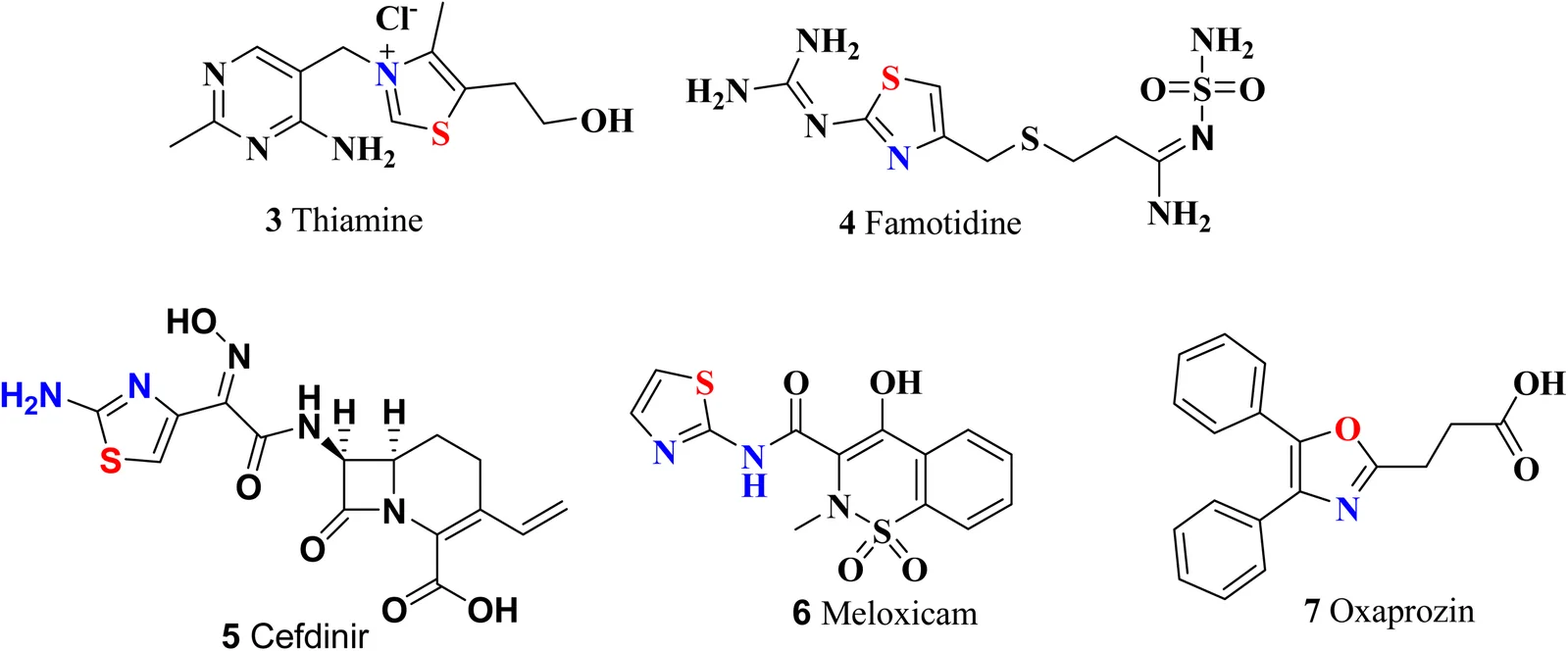
Representative biologically active molecules containing thiazole and oxazole rings.

Introducing an amino group at the C2 position of oxazole and thiazole gives the corresponding 2-aminooxazoles (8) and 2-aminothiazoles (9). The C2-amino group adds another site for hydrogen bonding and can be further modified, while the oxygen or sulfur atom in the ring contributes to differences in the electronic and physicochemical properties of these compounds. These structural features have made aminoazoles useful in both synthetic chemistry and medicinal chemistry. 2-Aminothiazoles have been studied widely as medicinal-chemistry scaffolds, while interest in 2-aminooxazoles has grown in connection with their structural similarity to 2-aminothiazoles and their potential use as bioisosteric alternatives.^3,6^
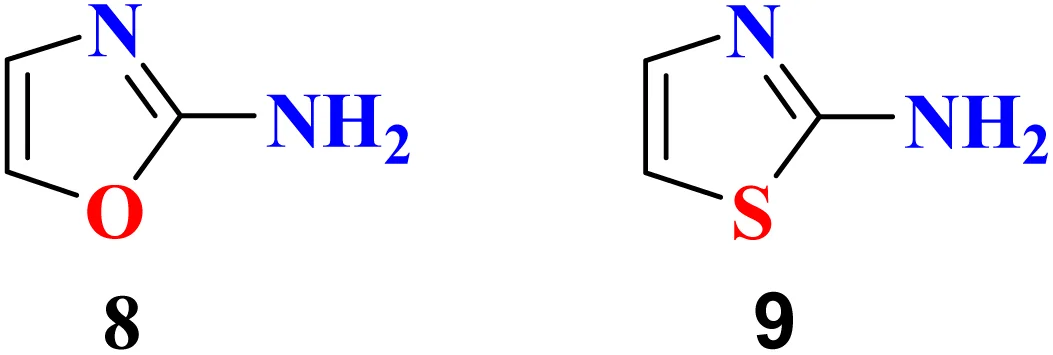


The biological and synthetic behaviour of these heterocycles can vary considerably with changes in substitution around the heterocyclic ring. In 2-aminothiazoles, for example, both the substituents attached to the ring and changes made to the exocyclic amino group have been used to modify molecular properties and biological activity. This flexibility has maintained interest in the scaffold in drug-discovery studies.^4,7^

In contrast to 2-aminothiazoles, 2-aminooxazoles are less extensively represented in marketed drugs, although sulfamoxole (10), a sulfonamide antibacterial containing a 2-aminooxazole moiety, provides an important example of the pharmacological utility of this relatively less explored scaffold. The close structural relationship between the two aminoazole classes has also made 2-aminooxazoles attractive as potential bioisosteric counterparts of 2-aminothiazoles. Replacement of sulfur by oxygen can modify molecular size, polarity, hydrogen-bonding characteristics, physicochemical properties, and biological behaviour, although the effect is dependent on the molecular framework and biological context rather than being uniformly favourable. Direct comparative studies have demonstrated changes in physicochemical properties and biological performance following O-for-S replacement, while selected antitubercular series have shown that 2-aminooxazoles can retain activity comparable to corresponding 2-aminothiazoles.^3,7,8^
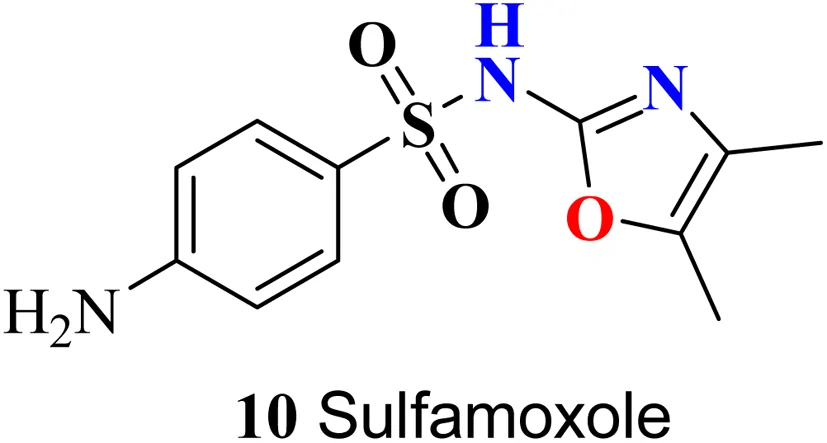


An important structural feature of 2-aminooxazoles and 2-aminothiazoles is substitution at the C4 and C5 positions, which gives regioisomers having the same heterocyclic framework but different positions of the substituent relative to the C2-amino group and ring heteroatom [Fig. 2]. For example, 2-amino-4-phenylthiazole (11) and 2-amino-5-phenylthiazole (12) represent two positional isomers in which the phenyl group occupies different positions on the ring. Related substitution has also been reported in 2-aminooxazoles, including 2-amino-4-phenyl-1,3-oxazole (13) derivatives.^9^ A change in the position of a substituent can modify the electronic environment, molecular shape, hydrogen-bonding interactions, and its accessibility to other parts of a molecule. These differences can be reflected in molecular recognition and biological activity. Studies of substituted 2-aminothiazoles have reported activity differences associated with both the substituent and its position, although the extent and direction of these effects vary between chemical series.^10,11^ The C4/C5 distinction is important for both the synthesis and biological evaluation of aminoazole derivatives. The available studies do not show a consistent preference for C4- or C5-substitution across different aminoazole series. It is therefore important to prepare the regioisomers selectively and confirm their structures before comparing their chemical or biological properties.

**Fig. 2:**
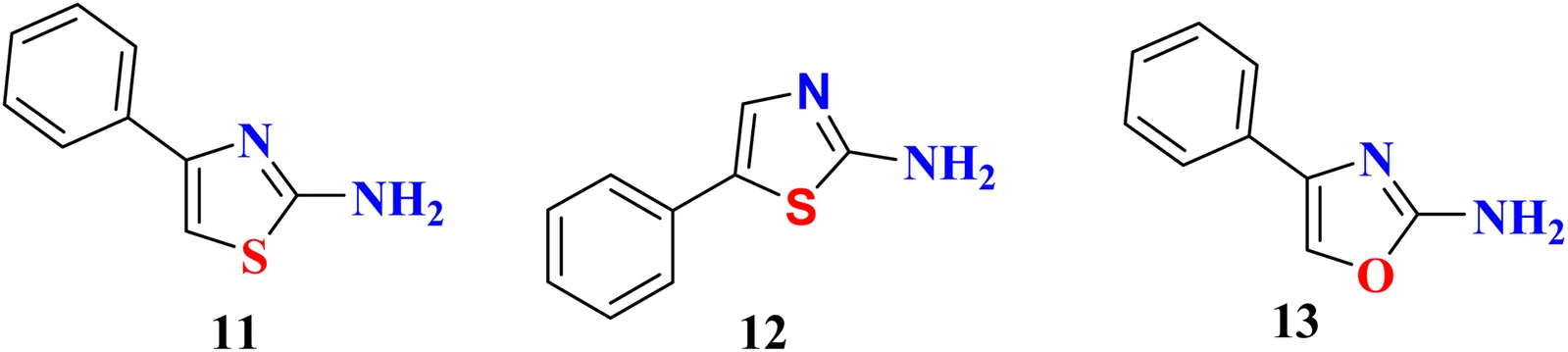
2-Aminoazole regioisomers at the C4 and C5 positions.

Selective preparation of these regioisomers is not always straightforward. The outcome can change with the starting material, substituent, and reaction conditions. Classical cyclization reactions, including the Hantzsch synthesis of thiazoles, have provided useful routes to these heterocycles. Other classical approaches, such as the Cook–Heilbron reaction, have also contributed to thiazole synthesis.^12–14^

Traditional procedures may require several reaction steps, prolonged heating, excess reagents, or hazardous solvents. Product purification can also involve chromatography. These limitations have encouraged the use of microwave and ultrasound irradiation, one-pot reactions, reduced-solvent procedures, heterogeneous catalysts, and continuous-flow methods. The use of one of these techniques does not automatically make a reaction greener. Solvent consumption, energy use, waste, catalyst recovery, and purification should also be taken into account.^14,15^

Computational studies provide another way to examine aminoazole chemistry. For reaction studies, calculations can help compare competing pathways and identify factors that may favour one regioisomer. Molecular modelling can also be useful when examining possible structure–activity relationships. Data-driven approaches provide an additional source of information, particularly when a sufficiently large experimental dataset is available. This is relevant to regioselective reactions because changes in the substrate or reaction conditions can alter the distribution of products.^16^ Computational results, however, need experimental support. Their reliability depends on the method used, the reaction model, and the quality of the experimental data available for comparison.

Previous reviews have covered different parts of oxazole and thiazole chemistry, including synthesis, medicinal applications, and biological activity. More recent work has also considered greener approaches to their preparation.^3,4,15,17^ These articles generally examine individual scaffolds or selected aspects of their chemistry. The relationship between C4/C5 regioisomerism, regioselective synthesis, biological activity, and sustainability has received less attention. This provides the basis for examining these aspects together in the present review. Here, we consider 2-aminooxazoles and 2-aminothiazoles together and discuss how changes in their structures can affect both their synthesis and biological behaviour. Recent developments in greener synthetic methods and process-oriented approaches are considered alongside computational studies that help in understanding regioselectivity and structure–activity relationships. Rather than assigning a general preference to a particular heteroatom or C4/C5 regioisomer, the available results are discussed in relation to the chemical series and biological system involved. We also point out areas where the available evidence remains limited, including direct comparisons of C4/C5 regioisomers, matched O/S analogues, experimental testing of computational predictions, and quantitative evaluation of the sustainability of synthetic procedures.

## 2 Structural and pharmacological relevance

2-Aminooxazoles and 2-aminothiazoles contain a five-membered heteroaromatic ring with oxygen or sulfur as the ring heteroatom and an exocyclic amino group at C2. The combination of these structural features gives aminoazoles a characteristic electronic environment and provides sites for further structural modification.^16–18^ The C2-amino group can participate in hydrogen bonding and can also be modified to introduce changes in the physicochemical and biological properties of the molecule. Aminoazole derivatives have consequently been explored as synthetic intermediates as well as in medicinal chemistry and drug design.^16,18^ Replacement of oxygen by sulfur further changes the electronic and polarizability characteristics of the ring and can influence hydrogen-bonding interactions and other non-covalent interactions with biological targets. These effects, however, depend on the complete molecular structure, including the C2-amino group and substituents at other ring positions.^16,18–21^

### 2.1 C4/C5 regioisomerism and structural consequences

Substitution at C4 and C5 gives regioisomeric 2-aminooxazoles and 2-aminothiazoles. These compounds have the same molecular formula but differ in the position of the substituent on the heteroaromatic ring. Changing the substitution site also changes its proximity to the C2-amino group and the ring heteroatom. This difference can affect steric effects, electronic distribution, hydrogen bonding, and molecular recognition. These differences are relevant to medicinal chemistry because even a small positional change can modify ligand–target interactions and produce measurable changes in structure–activity relationships. For example, SAR studies of 2-aminothiazoles have shown that substitution at the C4 position can strongly influence antimycobacterial activity, demonstrating that positional changes should be evaluated experimentally rather than treated as interchangeable structural variations.^18^ The nature of the ring heteroatom also contributes to these differences; replacement of sulfur with oxygen in matched 2-aminothiazole/2-aminooxazole series has been shown to modify physicochemical properties and retain or alter biological activity depending on the compound series.^8^ The combined influence of the ring heteroatom, C2-amino group and C4/C5 substitution pattern on the structural and physicochemical behaviour of these scaffolds is summarized in Table 1. Accordingly, C4 and C5 should be treated as distinct structural sites when establishing SAR, particularly when comparing regioisomeric compounds.

**Table 1:** Structural features and their influence on the properties and biological activity of 2-aminooxazoles and 2-aminothiazoles

Entry	Structural feature	Consequence	H-bonding/physicochemical properties	Pharmacological relevance	Ref.
1	Ring heteroatom (O/S)	Replacement of O by S changes the size, polarizability and electronic properties of the ring	Affects polarity, hydrophilicity, lipophilicity and intermolecular interactions	Differences in these properties can influence cellular uptake and biological activity	3 and 8
2	C2-amino group (–NH_2_)	The amino group is conjugated with the azole ring and contributes to its electronic properties	Acts mainly as a hydrogen-bond donor and participates in intermolecular interactions	Important for molecular recognition and binding to biological targets	3, 19, and 20
3	C4/C5 substitution	Moving a substituent from C4 to C5 changes its position relative to N3, O/S and the C2-amino group	The HBD/HBA count may remain unchanged, but the geometry and accessibility of interaction sites can differ. Crystal packing and other solid-state properties may also change	Different positioning of the substituent can affect binding interactions and biological response	19 and 20
4	Electronic effects of substituents	Electron-donating and electron-withdrawing groups change the electron density of the azole ring	Can affect the electronic environment of the amino group and ring heteroatoms, as well as protonation and reactivity	May influence target binding, potency and selectivity	3 and 21
5	Hydrogen bonding	The C2-NH_2_ group and ring N/O/S provide important sites for intermolecular interactions	C4/C5 substitution can change the orientation and accessibility of these sites without necessarily changing the number of H-bond donors or acceptors	Hydrogen bonding can contribute to target recognition and binding affinity	3 and 19
6	Hydrophilicity, lipophilicity and solubility	Ring heteroatom and substituent identity affect the balance between hydrophilic and hydrophobic character	Aminooxazole analogues have been reported to show higher hydrophilicity and water solubility than corresponding aminothiazoles in matched compounds	Solubility and lipophilicity influence membrane permeation and availability of the compound at the target site	8
7	Solid-state properties	Different regioisomers and heteroatom substitutions can lead to different crystal-packing arrangements and intermolecular contacts	These differences can affect melting behaviour, crystallinity and dissolution	May influence formulation and the amount of compound available for biological action	19
8	Pharmacophore orientation	C4/C5 substitution changes the relative position of the substituent and the C2-amino group with respect to the ring heteroatoms	This can change the way H-bonding, hydrophobic and electrostatic interactions are presented to the surrounding environment	May affect binding mode, affinity and selectivity	3
9	Pharmacological activity	Changes in the ring heteroatom, substitution pattern and electronic properties can produce different biological profiles	Physicochemical properties and molecular interactions both contribute to the observed activity	2-Aminothiazoles and 2-aminooxazoles have been investigated for antimicrobial, anticancer, enzyme-inhibitory and other pharmacological activities	3, 8, and 21

### 2.2 Pharmacological relevance and structure–activity relationships (more updated)

The biological activity of 2-aminooxazoles and 2-aminothiazoles is strongly influenced by the substitution pattern of the aminoazole framework. In particular, modification of the exocyclic amino group, substitution at C4/C5, and variation of the attached aryl or heteroaryl groups can produce substantial changes in biological activity. However, the effect of a given structural modification is often dependent on the chemical series and the biological system investigated.

Pieroni *et al.* investigated a series of *N*-substituted 2-aminothiazoles as antitubercular agents and demonstrated that modification of the exocyclic amino group could modulate activity within the investigated series. Selected N-substituted derivatives showed activity against *Mycobacterium tuberculosis*, illustrating the importance of this position for further structural optimization.^11^

Mjambili *et al.* further demonstrated the importance of substitution around the 2-amino-4-(2-pyridyl)thiazole scaffold in both antimycobacterial and antiplasmodial studies. A 2-pyridyl substituent at C4 was favourable in their reported series, while replacement with other pyridyl isomers resulted in reduced antimycobacterial activity. Substitution of the exocyclic amino group with a substituted phenyl group and an amide linker also contributed to the activity of several derivatives.^10^ The effect of substituent position was not identical in the two biological assays; for example, the positional preference of bromine substitution differed between the antimycobacterial and antiplasmodial series. These observations indicate that substituent effects should be interpreted in relation to both their position and the biological endpoint being measured.

The influence of the ring heteroatom has also been investigated through the replacement of sulfur in 2-aminothiazoles by oxygen. Azzali *et al.* examined matched 2-aminothiazole/2-aminooxazole pairs in an antitubercular series and found that several aminooxazole derivatives retained activity comparable to their thiazole counterparts, supporting the use of the aminooxazole ring as a bioisosteric replacement in the investigated scaffold.^6^ Importantly, the S → O replacement did not produce a uniform improvement in all physicochemical properties across the matched pairs, indicating that the consequences of this isosteric exchange depend on the molecular framework.

This point was further demonstrated by Juhás *et al.*, who systematically compared matched 2-aminothiazole and 2-aminooxazole derivatives. Replacement of sulfur by oxygen generally decreased the experimental log *k*′*w* values, corresponding to increased hydrophilicity in the aminooxazole series.^8^ Improved water solubility was also observed for several matched pairs; for example, the aminooxazole derivatives 12b and 15b showed approximately 413-fold and 59-fold higher solubility, respectively, than their corresponding thiazole analogues.^8^ However, the effect on antimycobacterial activity was not uniform. Some aminooxazole derivatives showed markedly improved activity, whereas other matched pairs exhibited comparable or lower activity than the corresponding thiazoles.^8^ The same study also showed that the substitution pattern of the attached heteroaromatic group could substantially influence activity, with pyridine-containing derivatives generally showing favourable activity in the investigated series.^8^ The Fig. 3 summarizes SAR observations reported by.^6,10,11^

**Fig. 3:**
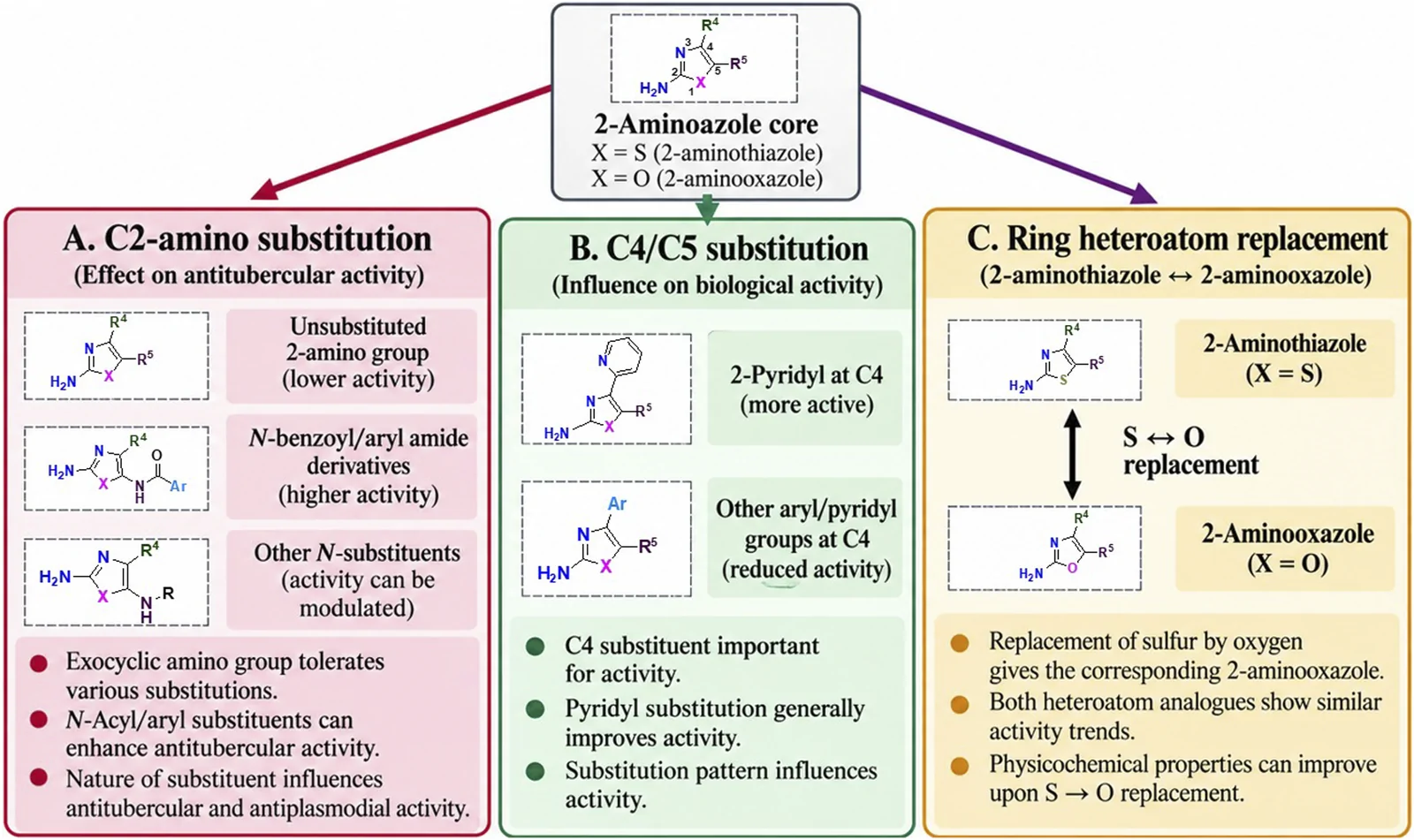
Selected structure–activity relationships of 2-aminoazoles reported in antitubercular, antimycobacterial and antiplasmodial studies. (A) Effect of modification at the exocyclic C2-amino group; (B) influence of C4 substitution and substituent pattern on biological activity; and (C) relationship between 2-aminothiazoles and 2-aminooxazoles resulting from S → O replacement.

### 2.3 Structural validation and interpretation

Reliable structural assignment is essential when closely related C4- and C5-regioisomers are discussed. For 2-aminoazoles, ^1^H and ^13^C NMR spectroscopy, supported by IR and HRMS, provides important evidence for confirming the proposed structures.^6^

However, one-dimensional NMR may not always be sufficient to distinguish closely related regioisomers. In such cases, 2D NMR techniques, particularly HMQC and HMBC, can establish long-range correlations between substituents and specific ring carbons. A recent study on regioselective thiazole synthesis demonstrated that these correlations provided clear structural assignments, which were further supported by single-crystal X-ray diffraction.^22^ Such structural confirmation is particularly relevant when different cyclization pathways can generate regioisomeric products. The importance of substitution pattern is also evident from biological studies of 2-aminothiazoles.

For example, Mjambili *et al.* reported that the 2-amino-4-(2-pyridyl) thiazole scaffold showed significant dependence of antimycobacterial activity on substitution at the amino group and the C4-linked pyridyl group.^10^ Similarly, comparison of 2-aminothiazoles with their 2-aminooxazole counterparts showed that replacement of sulfur by oxygen can maintain antitubercular activity while modifying physicochemical properties.^6^ These findings indicate that biological behaviour cannot be assigned solely to the C4 or C5 position without considering the complete substitution pattern and assay conditions. Therefore, experimental structural characterization should precede SAR interpretation, while DFT and molecular modelling can subsequently help explain electronic or binding differences.

Importantly, computational predictions should be regarded as supportive evidence and not as a substitute for experimental confirmation of the regioisomer. Thus, combining spectroscopic, crystallographic and biological evidence provides a more reliable basis for correlating regioselective structure with biological activity.

## 3 Classical and green synthetic strategies

Conventional methods for 2-aminooxazoles and 2-aminothiazoles have recently been improved with the introduction of greener and more selective methods. Classical methods still have value for the regioselective synthesis of the aminoazole framework from functionalized precursors. The practical utility of these methods is dependent on the substrate, as well as the efficiency of the regioselective cyclization and purification of the product. Therefore, advancements in selectivity and reduced consumption of reagents, solvents, and other reaction components should be viewed as complementary to the development of more classical chemistry, including strategies for the reformulation and improvement of existing chemical bonds.^23–27^

### 3.1 Limitations of conventional synthesis

Classical synthetic procedures have provided reliable access to aminoazole frameworks, but they often involve several operations from substrate preparation to product purification. For 2-aminooxazoles, Klug *et al.* noted that an established approach based on an isothiocyanate, β-keto azide and triphenylphosphine generally required reflux in dioxane followed by flash chromatography, with moderate to good yields depending on the substrate.^14^ Similar concerns arise in classical 2-aminothiazole synthesis, where α-haloketones and thiourea or related thioamides are commonly employed and the reaction can involve halide-containing by-products and additional processing.^12,28^ These limitations do not diminish the synthetic value of the classical reactions, but they provide a clear reason to examine reaction time, solvent use, waste generation and purification alongside isolated yield.

### 3.2 Classical synthetic approaches (recent)

The synthesis of aminoazoles has been developed through several classical cyclization reactions. For 2-aminothiazoles (19), the Hantzsch synthesis is one of the commonly used methods and involves the reaction of α-halocarbonyl compounds (14) with thiourea (15) or substituted thioureas (17). The method is simple and can accommodate a range of substrates.^4,12,20^ A representative Hantzsch-type cyclization and related classical routes to 2-aminothiazoles are shown in Scheme 1.

**Scheme 1:**
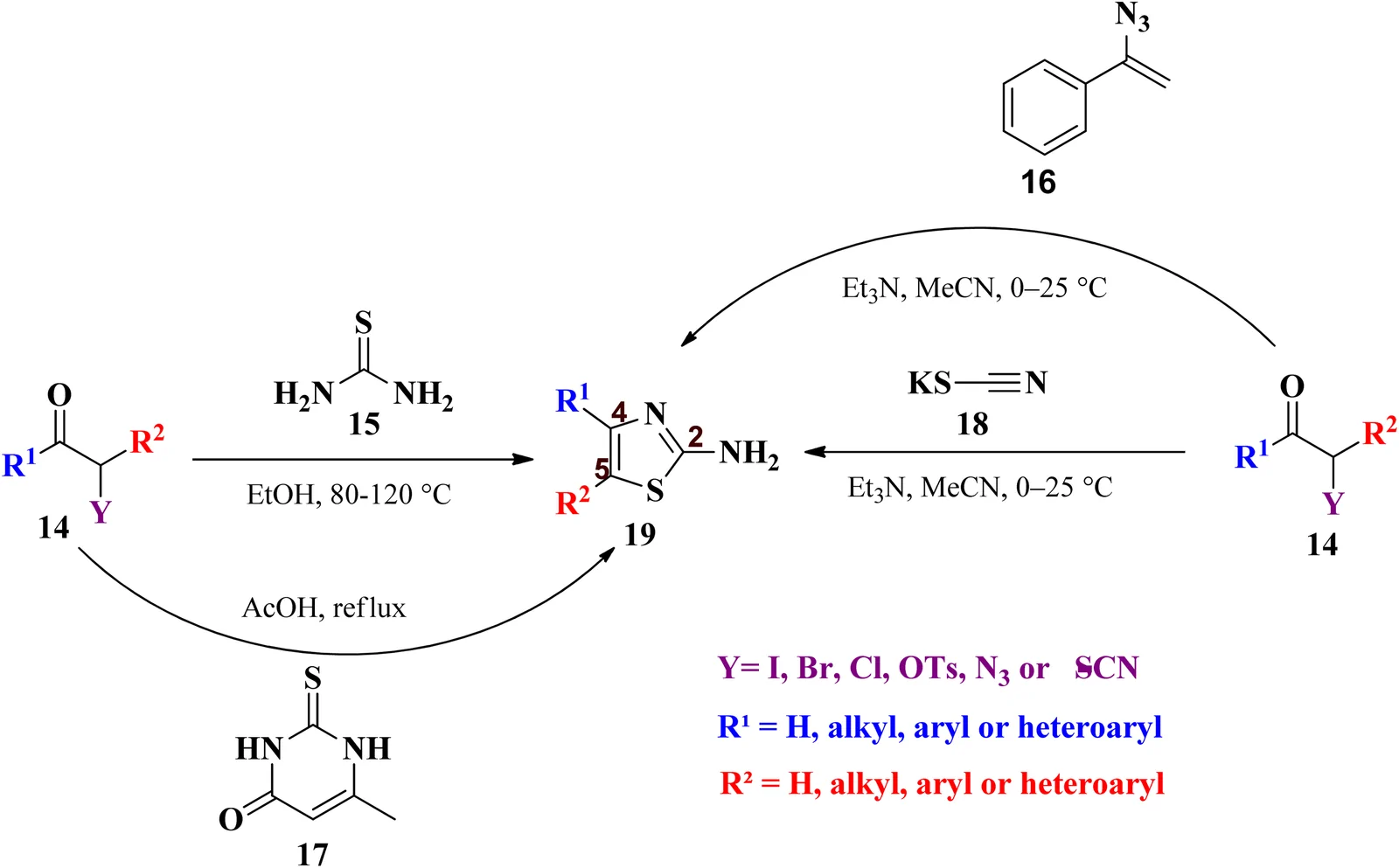
Hantzsch and related classical cyclization routes to 2-aminothiazoles.

The Cook–Heilbron synthesis provides another classical route to thiazoles and is particularly useful for the preparation of 5-aminothiazoles (21). In this approach, α-aminonitrile-derived substrates (20) undergo reactions with carbon disulfide, dithioacids, or related sulfur-containing reagents.^13,15^ The general reaction pattern of the Cook–Heilbron synthesis is given in Scheme 2.

**Scheme 2:**
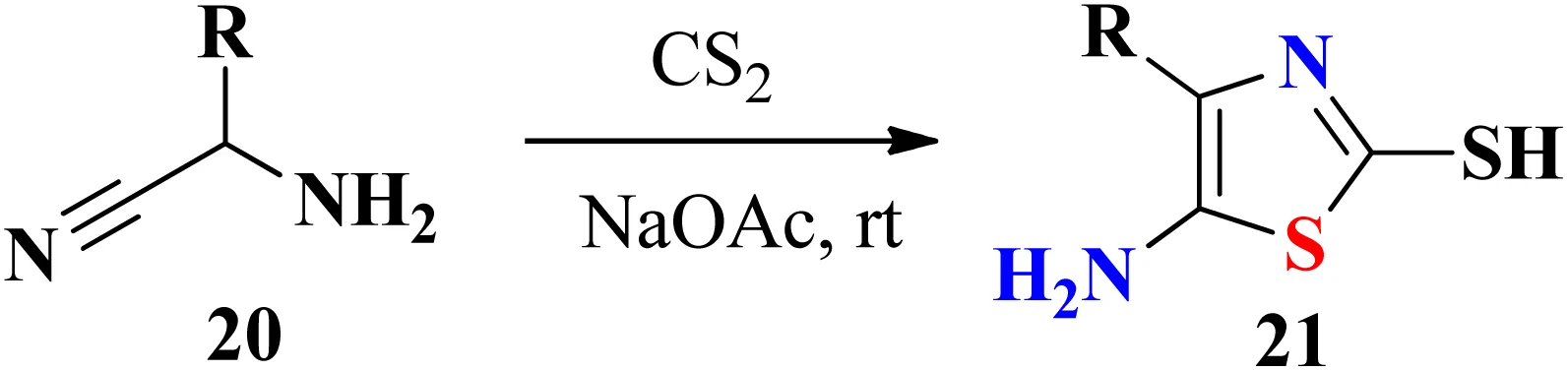
Cook–Heilbron synthesis of 5-aminothiazoles.

The preparation of 2-aminooxazoles has followed several different cyclization routes because suitable oxygen-containing precursors can be approached in more than one way. Reported methods include reactions involving α-azidoketones (22), α-bromoketones (26), isothiocyanates (24), and urea derivatives (25).^14^ Some representative classical routes to 2-aminooxazoles are summarized in Scheme 3.^17,27^ These methods provide useful access to aminoazole frameworks, but they may also require activated starting materials, several reaction steps, heating, or conventional purification. These limitations have encouraged the development of simpler, more selective, and environmentally preferable synthetic methods, which are discussed in the following sections.

**Scheme 3:**
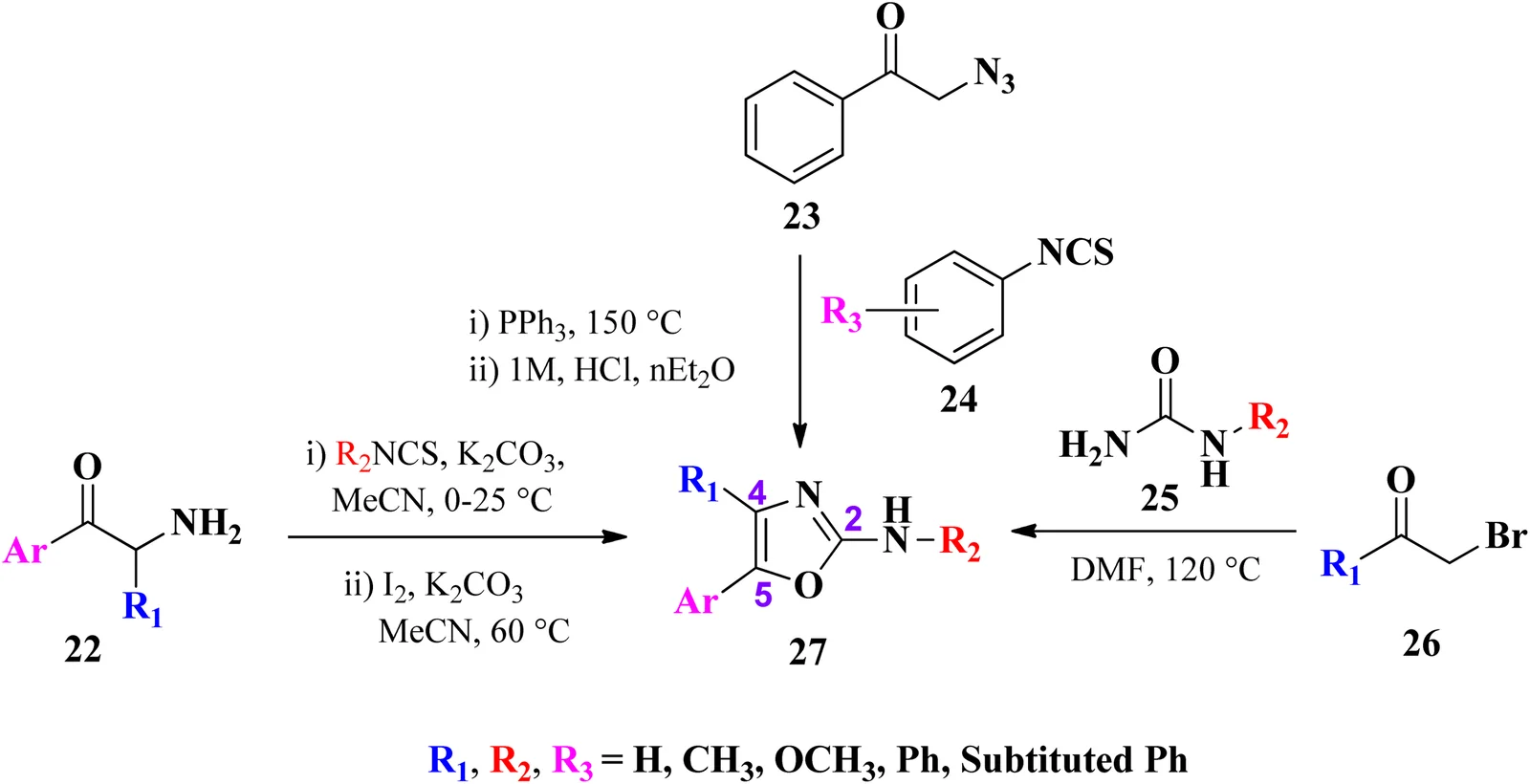
Representative classical synthetic routes to 2-aminooxazoles.

### 3.3 Environmental and process considerations

The environmental performance of these reactions cannot be judged from yield alone. Solvent consumption, reagent excess, energy requirement, waste formation, catalyst loading and the work-up procedure all contribute to the overall process burden. Green-chemistry metrics such as atom economy, *E*-factor, reaction mass efficiency and process mass intensity provide complementary ways of assessing these factors.^29^ Solvent use deserves particular attention because solvents frequently represent a major part of the material input and environmental impact of organic and pharmaceutical synthesis.^30–32^ Therefore, a shorter reaction, reduced solvent volume or simpler isolation can be meaningful improvements, but a method should be described as greener only when these advantages are considered together with the complete process. Representative classical and emerging green approaches for the synthesis of 2-aminooxazoles and 2-aminothiazoles, together with their key synthetic and process features, are summarized in Table 2.

**Table 2:** Classical and green synthetic approaches to 2-aminooxazoles and 2-aminothiazoles

Entry	Approach	Representative reaction/system	Important feature	Process advantage/limitation	Ref.
1	Hantzsch synthesis	α-Haloketone + thiourea/thioamide	Established route to 2-aminothiazoles	Reliable and versatile; requires prefunctionalized α-haloketones	12
2	Cook–Heilbron synthesis	α-Aminonitrile + sulfur-containing reagent	Classical route to 5-aminothiazoles	Useful access to amino-substituted thiazoles	13
3	Conventional 2-aminooxazole synthesis	β-Keto azide + isothiocyanate + PPh_3_	Provides highly functionalized 2-aminooxazoles	Reflux conditions and flash chromatography may be required	14
4	One-pot oxidative synthesis	Ketone + thiourea, I_2_/DMSO	Avoids preformation of α-haloketones	Fewer preparative steps; moderate–good yields	31
5	Microwave-assisted synthesis	β-Keto azide + isothiocyanate + PPh_3_	150 °C, 5 min	47–87% yields; simple precipitation without chromatography	14
6	Aqueous multicomponent synthesis	Isocyanide + amine + sulfur + 2′-bromoacetophenone	One-pot formation of 2-aminothiazoles	Water as medium; chromatography-free isolation; good atom economy	32
7	Green-process evaluation	*E*-factor, PMI, RME, atom economy	Quantitative assessment of process efficiency	Yield alone does not describe overall greenness	29

### 3.4 Evolution from classical to green synthesis

The evolution of aminoazole synthesis has involved changes not only in reagents and catalysts but also in energy input, reaction media, and process design. Microwave irradiation, ultrasound, one-pot reactions, and aqueous or solvent-minimized systems have been explored as alternatives to conventional procedures.^33,34^

A representative example is the microwave-assisted synthesis of 2-aminooxazoles reported by Klug *et al.*, which afforded suitable substrates in 47–87% yield at 150 °C within 5 min, with isolation by precipitation and without chromatographic purification. However, electronically deactivated and sterically demanding substrates remained challenging, indicating that the advantages of microwave irradiation are still influenced by the intrinsic reactivity of the substrate.^14^ For comparison of the synthetic methods reported in the literature, the studies included in this review were classified according to their principal synthetic approach [Fig. 4].^17,20^ Classical Hantzsch multicomponent reactions accounted for the largest group (36 studies), followed by conventional transition-metal-catalyzed methods (21 studies). Microwave-assisted methods (7 studies) and ultrasound-assisted methods (5 studies) were also reported, whereas aqueous/solvent-free and one-pot multicomponent approaches were less frequently investigated, with two and one studies, respectively. The frequency of a particular method, however, does not necessarily reflect its sustainability or regioselectivity. These aspects depend on several factors, including solvent and energy requirements, waste generation, catalyst loading, purification, and the overall efficiency of the process.^35^ The comparison also highlights an important aspect of aminoazole synthesis: improving reaction efficiency does not always guarantee control over regioselectivity. This is particularly relevant for reactions in which C4- and C5-functionalization or cyclization pathways compete, and forms the basis for the discussion of green regioselective synthesis in the following section.

**Fig. 4:**
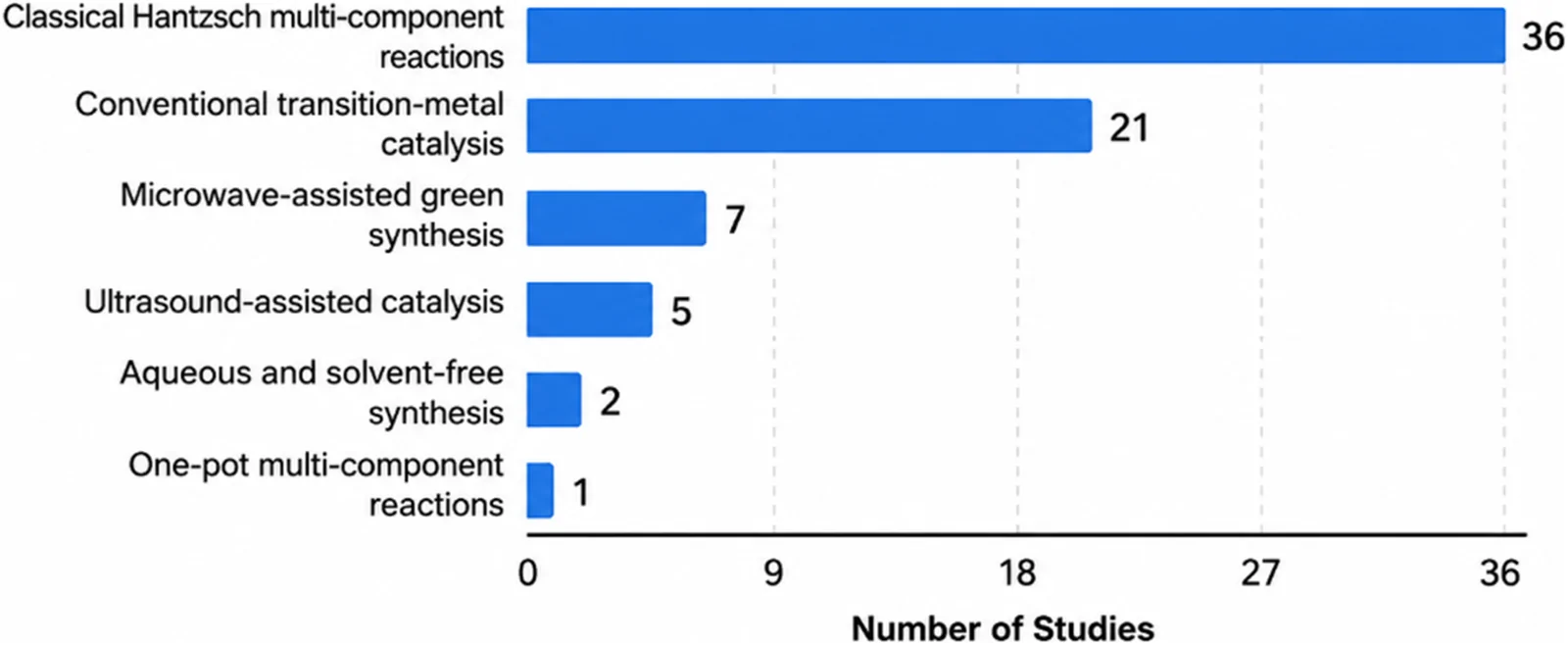
Distribution of literature reports on the synthesis of 2-aminooxazoles and 2-aminothiazoles according to the principal synthetic approach.

## 4 Green regioselective synthesis

Recent work on 2-aminooxazoles and 2-aminothiazoles has focused not only on the formation of the heterocyclic ring but also on controlling the position at which cyclization occurs. This issue is particularly relevant for unsymmetrical substrates, where more than one cyclization pathway may be possible and can lead to different regioisomers or, in some cases, rearranged products. Therefore, an efficient synthetic method should ideally provide good regioselectivity while also minimizing solvent use, reaction time, energy consumption, waste, and purification requirements. Several methods have been explored to make aminoazole synthesis faster and simpler. These include microwave irradiation, aqueous or solvent-free reactions, ultrasound-assisted methods, one-pot procedures, and continuous-flow processing. Their environmental advantage cannot be judged from the reaction technique alone. Substrate scope, catalyst loading, energy use, work-up, and scalability also affect the overall performance of the process.

### 4.1 Regioselective cyclization and mechanistic control

Regioselectivity in aminoazole synthesis originates from the competition between alternative reaction pathways leading to different constitutional products. The outcome may be influenced by the electronic character of the electrophilic precursor, the nucleophilicity and substitution pattern of the thiourea or urea component, steric interactions, conformational effects, and the reaction environment. Therefore, the formation of a particular C4- or C5-substituted aminoazole should be regarded as a substrate- and condition-dependent outcome rather than a universal positional preference.^20,36^

A particularly relevant study was reported by Aggarwal and co-workers for the reaction of α-tosyloxyacetophenones with *N*-substituted thioureas. Depending on the substituents present on both reaction partners, the reaction afforded either 2-aminothiazoles or 2-imino-2,3-dihydrothiazoles. The authors proposed that the observed regioselectivity was governed by the relative reactivity of the competing reaction sites and supported the proposed mechanism using DFT calculations. The study therefore provides an important example of how substituent effects can alter the preferred cyclization pathway.^36^

This observation is particularly important for aminoazole chemistry because the identity of the product cannot always be predicted simply from the substitution pattern of the starting material. Electronic effects can alter the relative electrophilicity of competing carbon centres, whereas steric effects can influence the orientation of the nucleophile during cyclization. The reaction medium can further modify these factors by changing protonation states, hydrogen bonding, and intermediate stability.

More recent work by Aggarwal and co-workers provides an even clearer illustration of competing pathways. The reaction of unsymmetrical α-bromo-1,3-diketones (23) with *N*-substituted thioureas (24) produced 2-(*N*-arylamino)-5-acyl-4-methylthiazoles (25) (26) in some cases, whereas other substrates underwent C–N acyl migration to produce rearranged 2-(*N*-acylimino)-3-*N*-aryl-4-methylthiazoles (27) (28) [Scheme 4]. In some combinations, mixtures were obtained. The authors used multidimensional NMR and X-ray crystallography to establish the structures of the products.

**Scheme 4:**
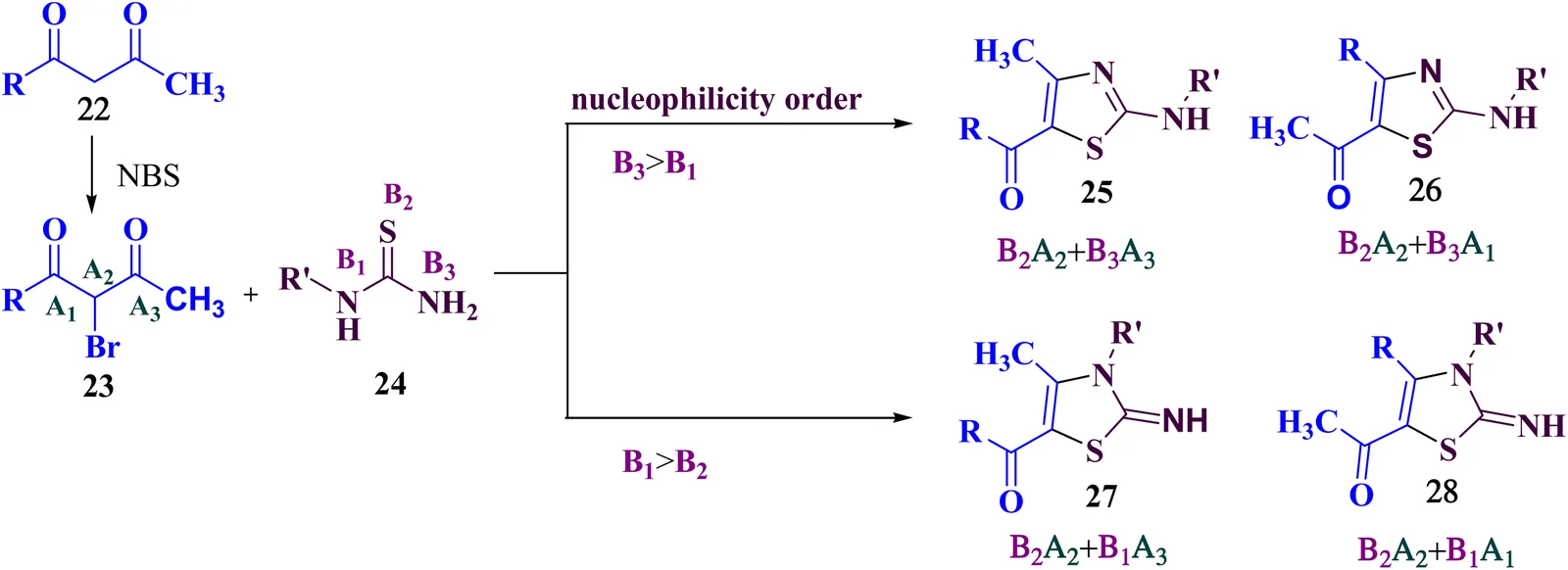
Competing cyclization and rearrangement pathways in the reaction of unsymmetrical α-bromo-1,3-diketones with N-substituted thioureas, illustrating the influence of substrate structure on regioselective aminoazole formation.^22^

Another useful approach involves the direct formation of 2-aminothiazoles (30) from ketones (29) and thiourea (15). Ghodse and Telvekar reported that substituted ketones react with thiourea in the presence of aqueous NaICl_2_ in THF under reflux to furnish 2-aminothiazoles in good to excellent yields [Scheme 5]. In this approach, the reactive α-halogenated intermediate is generated *in situ*, avoiding its separate preparation and isolation and thereby simplifying the synthetic sequence.^37^ This type of *in situ* activation is particularly relevant to green synthesis because generation and consumption of a reactive intermediate can be combined into a single operation, reducing the need for intermediate isolation.

**Scheme 5:**
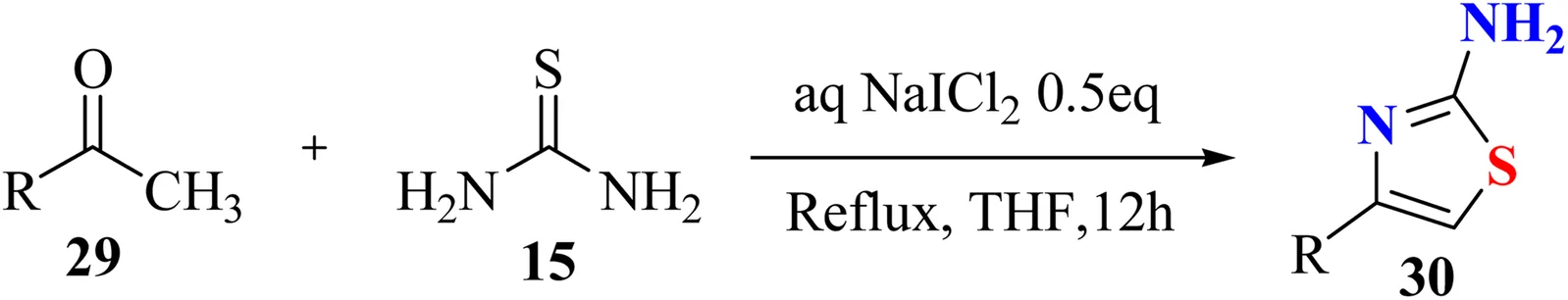
Direct synthesis of 2-aminothiazoles from ketones and thiourea using aqueous NaICl_2_.^37^

### 4.2 Green and process-intensified regioselective synthesis

In aminoazole synthesis, regioselectivity is influenced not only by the starting materials but also by using the reaction conditions used for cyclization. The reaction medium, temperature, heating method, and mode of energy transfer will affect both reaction efficiency and product distribution. Microwave irradiation, ultrasound, aqueous media, one-pot transformations, mechanochemistry, and continuous-flow processing have therefore been explored as alternatives to conventional reaction conditions. Microwave irradiation, in particular, has been widely used to accelerate organic and heterocyclic reactions. Polshettiwar and Varma discussed its application in aqueous and other environmentally preferable media and showed that rapid microwave heating can substantially reduce reaction times for a variety of organic transformations.^33^

An example relevant to 2-aminooxazole (33) synthesis was reported by Klug and co-workers using microwave [Scheme 6]. The reaction was carried out at 150 °C for 5 min, and the products were generally obtained by simple precipitation, without recrystallization or flash chromatography. Moderate to good yields were obtained for a range of substrates. The short reaction time and simple product isolation are useful features of this method. However, electronically deactivated and sterically demanding substrates showed poorer performance. Thus, rapid microwave heating does not necessarily overcome limitations arising from the intrinsic reactivity of the substrate.^14^

**Scheme 6:**
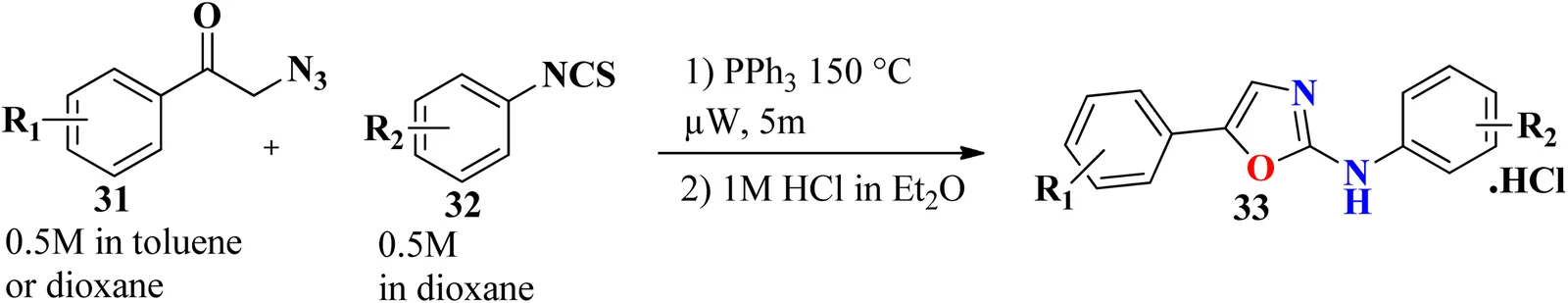
Microwave-assisted synthesis of 2-aminooxazoles.^14^

Ultrasound can improve reaction efficiency in different ways. The mixing and mass transfer generated by cavitation can speed up product formation in some reactions. Borah and Chowhan have recently reported a review on the ultrasound-assisted transition metal free reactions for the synthesis of bioactive heterocycles, and focused on areas of improvement in reaction time and efficiency.^38^

The use of aqueous conditions can be particularly advantageous for the synthesis of aminoazole compounds. Under these conditions, reactive intermediates can be generated and consumed in the same reaction vessel. For example, Ghodse and Telvekar recently reported the direct synthesis of substituted 2-aminothiazoles in aqueous NaICl_2_ from the corresponding ketones and thiourea, where the more reactive α-halothiazole intermediates are formed *in situ* and used for cyclization. The above-mentioned methodology simplifies the synthesis and reduces the number of steps required.^37^

Although the improved reaction times and yields do not always guarantee improved regioselectivity. For example, using different reaction conditions can influence the relative formation of C4- and C5-regioisomers. Therefore, in addition to improved yields and shorter reaction times, it should be established that the desired regioisomer is preferentially formed.

Continuous-flow chemistry enables even greater manipulation of reaction conditions and is especially useful for reactions which involve reactive intermediates. In flow reactors, variables including mixing and reagent concentrations can be precisely controlled. Rogers and Jensen discussed how continuous-manufacturing has the potential to reduce solvent consumption, isolation of intermediates and waste when the entire process is designed appropriately.^39^

An example relating to the synthesis of aminoazoles was reported by Lu and colleagues in 2025. They described a process for the continuous-flow α-bromination of aldehydes using bromine and investigated flow-reactor mono- and dibromination [Scheme 7]. The generated brominated intermediates were further elaborated in a two-step continuous-flow synthesis to generate 2-aminothiazoles. The researchers also disclosed a continuous process for the generation of a 2-aminothiazole Hep3B inhibitor [Scheme 7], which consisted of the three steps of α-bromination, formation of 2-aminothiazole and a final condensation step.^40^

**Scheme 7:**
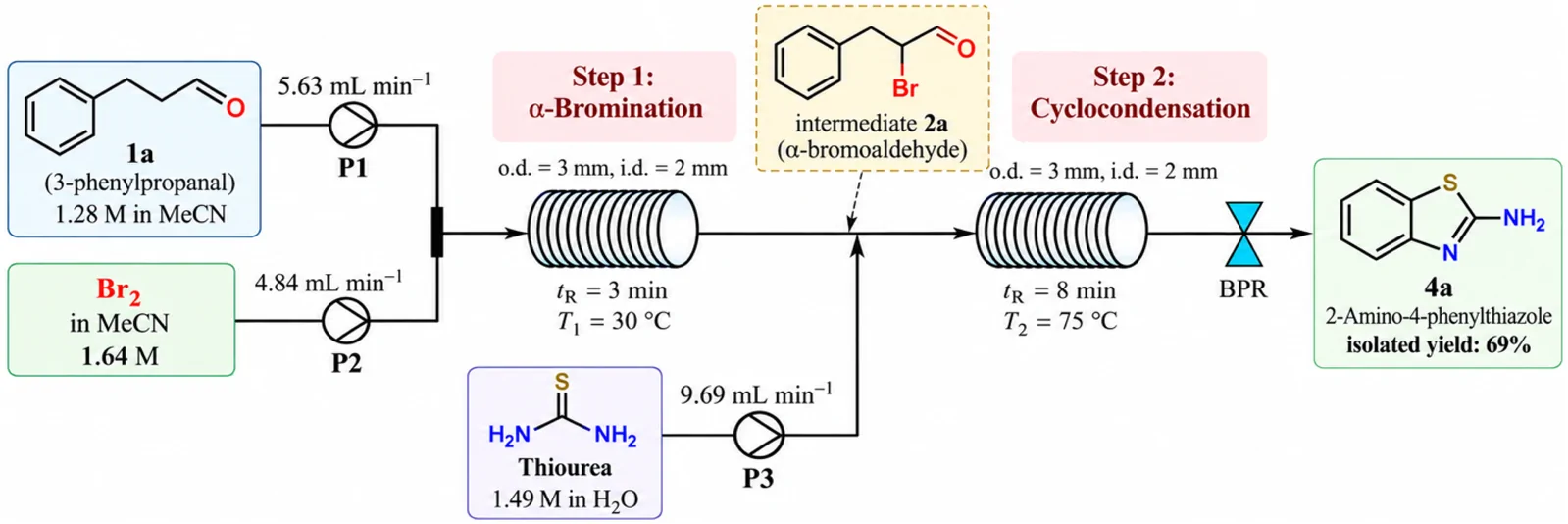
Continuous-flow synthesis of 2-aminothiazoles involving *in situ* α-bromination of aldehydes.^40^

While some of the improvements in regioselectivity in aminoazole syntheses may be attributed to the overall advances in green and intensified processes, it is prudent to consider all factors such as solvent and energy consumption and the overall number of synthetic steps in addition to regioselectivity, yield and waste.

### 4.3 Recent aminoazole examples and green synthetic opportunities

Recent work has explored ways to simplify aminoazole synthesis. Reactive intermediates can sometimes be generated *in situ* and taken directly into the cyclization step. One-pot reactions, microwave heating, and continuous-flow processing have also been used to reduce separate reaction and isolation steps. Such methods can reduce the need for intermediate isolation and, in some cases, shorten the reaction and purification steps.

For 2-aminooxazoles, Klug and co-workers reported a microwave-assisted method in which the reaction was completed within a few minutes at 150 °C. The products were generally obtained in moderate to good yields by simple precipitation, without recrystallization or flash chromatography. The method was less effective with some electronically deactivated and sterically demanding substrates, showing that the outcome still depends on the nature of the substrate.^14^

A similar simplification was reported for 2-aminothiazoles by Ghodse and Telvekar. They converted ketones and thiourea directly into substituted 2-aminothiazoles using aqueous sodium dichloroiodate (NaICl_2_). The α-haloketone required for cyclization is generated in the reaction mixture, so its separate preparation and isolation are avoided. Although the reaction was carried out in THF under reflux, combining intermediate formation and cyclization in a single operation reduces the number of steps involved in the synthesis.^37^

Bodireddy and co-workers reported another one-pot approach based on the sequential formation of the reactive intermediate and the thiazole ring. In their method, readily available aralkyl ketones (34) undergo α-bromination with *N*-bromosuccinimide in lactic acid, followed by reaction with thiourea to give the corresponding 2-aminothiazoles (35) [Scheme 8]. The reaction proceeds at 90–100 °C within a relatively short time, with lactic acid serving as both the reaction medium and promoter. Since the α-bromoketone is generated and consumed in the same reaction mixture, its separate isolation is avoided. The products were obtained without column chromatography, making the method a useful example of combining precursor formation and heterocycle synthesis in a single operation.^41^

**Scheme 8:**
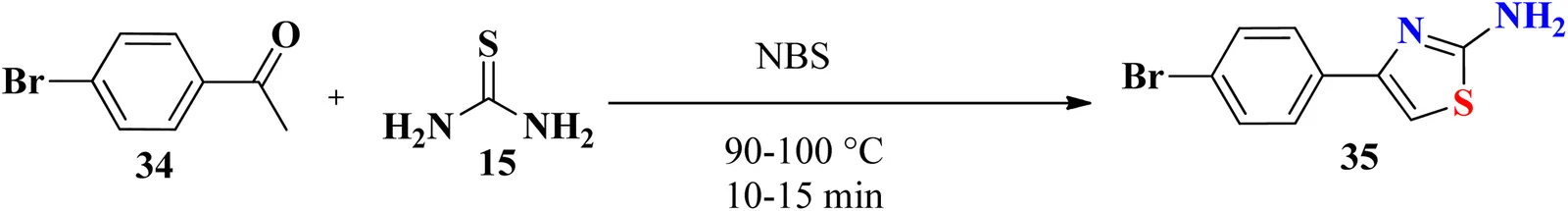
Lactic-acid-mediated tandem one-pot synthesis of 2-aminothiazoles through *in situ* regioselective α-bromination followed by thiourea cyclization.^41^

More recently, continuous-flow processing has provided another route toward improved control of reactive intermediates. Lu and co-workers investigated continuous-flow α-bromination followed by 2-aminothiazole formation, demonstrating the potential of controlled residence time, mixing, and heat transfer for improving the handling of reactive intermediates. Such approaches are attractive for scale-up because reaction and intermediate-generation steps can be integrated while reducing the accumulation of unstable intermediates.^40^

A further recent example is the molecular-sieve/I_2_-catalysed tandem synthesis of substituted 2-aminothiazoles reported by Serdaroğlu and co-workers. The study combines an environmentally oriented synthetic protocol with DFT calculations and biological evaluation, illustrating the increasing integration of synthesis, computation, and activity assessment within a single study. Importantly, computational analysis in such work can help interpret reaction pathways and molecular properties, but calculated preferences should be regarded as hypotheses that require experimental verification.^42^

The studies considered here show that no single approach addresses all of the challenges associated with green aminoazole synthesis. Microwave irradiation is effective for reducing reaction time, whereas one-pot and *in situ* procedures mainly simplify intermediate handling and product isolation. Aqueous media and continuous-flow methods provide additional opportunities to reduce solvent use and improve control over reaction conditions, but their advantages depend on the specific substrate and reaction sequence. Importantly, improvements in yield or reaction time do not necessarily correspond to better regioselectivity or lower environmental impact. A meaningful comparison therefore requires consideration of the complete process, including solvent and energy use, waste, purification, catalyst requirements, scalability, and the ratio of desired to competing regioisomers. From this perspective, the most useful developments are those in which process efficiency and environmental improvements are achieved without sacrificing reliable regioselective formation of the target aminoazole. Table 3. Shows green and process-intensified approaches relevant to the synthesis of 2-aminooxazoles and 2-aminothiazoles, highlighting their implications for reaction control and regioselective synthesis.

**Table 3:** Representative green and process-intensified approaches relevant to the synthesis of 2-aminooxazoles and 2-aminothiazoles

Entry	Strategy	Representative reaction/system	Green or process feature	Relevance to regioselective synthesis	Limitation	Ref.
1	Microwave irradiation	Isothiocyanate + β-keto azide → 2-aminooxazole	Rapid heating; 150 °C, 5 min; simple precipitation; no recrystallization or flash chromatography	Rapid reaction completion may limit the duration of competing processes	Substrate-dependent performance; some electronically deactivated or sterically demanding substrates show poorer conversion or mixtures	14
2	*In situ* oxidative activation	Ketone + thiourea → 2-aminothiazole using aqueous NaICl_2_	Avoids separate preparation and isolation of α-halogenated intermediates	Simplifies reactive-intermediate generation and reduces the number of isolated steps	THF/reflux conditions; solvent and energy requirements remain significant	37
3	Lactic-acid-mediated tandem process	Ketone → *in situ* α-bromination → thiourea cyclization → 2-aminothiazole	One-pot operation; lactic acid as reaction medium/promoter; avoids isolation of α-bromoketone; short reaction time	Integrates activation and cyclization in a single sequence	Requires 90–100 °C; substrate scope and scalability need to be considered	41
4	Continuous-flow synthesis	Flow α-bromination followed by 2-aminothiazole formation	Controlled residence time, mixing and heat transfer; reduced batch handling; enables multistep flow processing	Controlled generation and residence time of reactive intermediates may facilitate improved reaction control	Requires specialized equipment and process optimization	40
5	Molecular-sieve/I_2_-catalysed tandem synthesis	Tandem cyclization to substituted 2-aminothiazoles	Catalyst-assisted process using molecular sieve/I_2_; incorporates green-synthesis principles; computational and biological evaluation included	Provides an opportunity to connect reaction conditions with mechanistic/computational analysis; experimental regioselectivity still requires careful evaluation	Computational predictions and product preferences require experimental validation; broader substrate comparisons are needed	42
6	Ultrasound irradiation	Ultrasound-assisted heterocycle synthesis	Alternative energy input; can enhance mixing and mass transfer and reduce reaction times in suitable systems	May improve reaction efficiency through enhanced mass transfer and localized activation	Direct aminoazole-specific regioselectivity data remain limited	34

### 4.4 DFT- and AI/ML-assisted regioselectivity and remaining challenges

Experimental optimization alone can become inefficient when structurally similar products or competing reaction pathways are possible. Computational chemistry provides an additional means of examining why one pathway may be favoured over another. Depending on the mechanistic problem, DFT calculations can be used to evaluate the relative stability of intermediates or transition states and to examine electronic, charge-distribution, orbital and steric factors that influence the reaction outcome. Computational approaches have increasingly been incorporated into regio- and site-selectivity prediction, particularly through the combination of quantum-chemical descriptors and data-driven models.^16^

A directly relevant example in aminoazole chemistry is the work of Aggarwal and co-workers on the reaction of α-tosyloxyacetophenones with *N*-substituted thioureas. The authors observed that the ratio of 2-aminothiazoles and 2-imino-2,3-dihydrothiazoles changed with the substituents on the reaction partners. DFT calculations at the B3LYP/6-31G** level was used to compare the relative energies of protonated thiourea forms involved in the proposed mechanism, providing a mechanistic explanation for the experimentally observed product distributions.^36^

A more recent study further illustrates the complexity of competing pathways in aminoazole synthesis. Aggarwal and co-workers reported that reactions of unsymmetrical α-bromo-1,3-diketones with *N*-substituted thioureas can provide regioselective thiazoles as well as rearranged products, depending on the substrate and reaction conditions. Product structures were established using spectroscopic methods and, for selected compounds, X-ray crystallography. Such examples emphasize the need to consider alternative reaction pathways rather than assuming a single universal regioselective outcome.^22^

These examples illustrate an important role for computation in aminoazole chemistry: rather than simply predicting which product should form, computational analysis can be used to connect experimentally observed selectivity with plausible electronic and mechanistic factors. For future aminoazole studies, similar analysis could be extended to competing C4/C5 regioisomeric pathways by examining relevant transition states, intermediate stability, charge distribution, orbital interactions and steric effects. Such calculations would provide mechanistic hypotheses that can then be tested experimentally.

Machine learning offers a complementary strategy for handling larger reaction datasets. Recent computational studies have explored the use of molecular representations, quantum-chemical descriptors and experimental selectivity data for regio- and site-selectivity prediction.^16^ Importantly, ML–DFT integration has also been demonstrated for other classes of organic reactions. Guan and co-workers developed an approach in which machine-learning predictions were combined with DFT reaction modelling, with additional DFT calculations being triggered for predictions associated with greater uncertainty. Although their study focused on SNAr regioselectivity rather than aminoazole chemistry, it provides a useful methodological precedent for combining data-driven prediction with quantum-chemical analysis.^43^

For 2-aminooxazoles and 2-aminothiazoles, a future AI/ML model could therefore be developed using experimentally measured regioisomer distributions together with molecular, electronic and steric descriptors and reaction parameters such as solvent, temperature, catalyst or reagent and reaction time. A useful dataset should contain the substrate structure, reaction conditions, reaction yield and, most importantly, quantitative regioisomeric ratios established by appropriate analytical or structural methods such as NMR, HPLC/LC-MS or X-ray crystallography where applicable. Isolated yield alone is not sufficient to describe regioselectivity because a high overall yield does not establish the relative amounts of competing regioisomers.

A major research gap is the limited number of studies that compare C4- and C5-regioisomeric outcomes under identical or closely comparable conditions. Many reported methodologies demonstrate the successful synthesis of a particular regioisomer but do not provide quantitative information on the competing product. This limits the availability of consistent training data and consequently restricts the development and generalizability of AI/ML models. Computational predictions may also become less reliable when substrates fall outside the chemical space represented in the training dataset. Experimental validation is therefore essential when computational models are used to guide the synthesis of new substrates.^16^

The computational and predictive aspects should also be considered together with the sustainability of the synthetic process. Green chemistry assessment should include solvent use, energy demand, waste generation, purification requirements and catalyst recovery rather than relying on isolated yield alone. Continuous processing, for example, can provide opportunities to reduce waste and improve process efficiency, while microwave and ultrasound activation have been investigated as alternative approaches for improving reaction efficiency.^33,34,39^

Importantly, a greener reaction does not necessarily provide high regioselectivity, just as high regioselectivity does not automatically indicate a sustainable process. Recent aminoazole examples involving microwave-assisted synthesis and continuous-flow processing illustrate how reaction time, energy input and process configuration can be considered alongside synthetic performance.^14^

A useful future strategy would therefore integrate mechanistic analysis, DFT calculations, AI/ML prediction, experimental validation and green-process evaluation within an iterative framework. Such an approach could help establish a more reliable relationship between substrate structure, reaction conditions, regioselectivity and sustainability in aminoazole synthesis. A proposed DFT–AI/ML-assisted workflow for aminoazole regioselectivity prediction and experimental validation is shown in Fig. 5.^16,36,43^

**Fig. 5:**
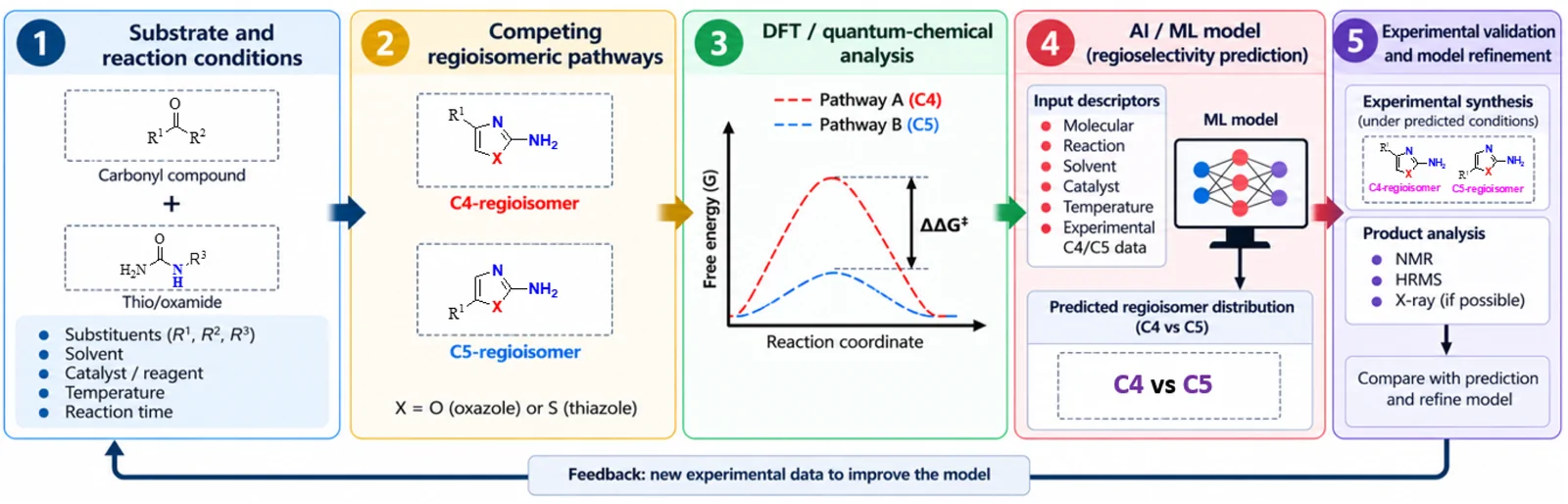
Proposed DFT–AI/ML-assisted workflow for regioselectivity prediction and experimental validation in 2-aminooxazole and 2-aminothiazole synthesis.

## 5 Biological importance

The biological activities of 2-aminooxazole and 2-aminothiazole derivatives are influenced by the nature and position of heteroatoms, functional-group substitution, electronic properties, hydrogen-bonding interactions, and molecular conformation. These aminoazole frameworks have been explored in a range of medicinal applications, including cancer, infectious diseases, inflammation, neurodegenerative disorders, and metabolic diseases. The biological evidence is not uniform. Some studies are based on biochemical or cellular assays, whereas others include computational analyses or selected *in vivo* experiments. Results from such different experimental settings cannot always be compared directly using numerical potency values. The biological target, assay conditions, and extent of experimental validation need to be considered when interpreting these results. This is especially relevant for heteroatom replacement and C4/C5 regioisomerism, where comparable biological studies are still relatively limited.^8,44^

### 5.1 Anticancer activities

The 2-aminothiazole scaffold is a common structural motif in kinase-directed medicinal chemistry. Deng *et al.* reported a series of 2-aminothiazole derivatives as checkpoint kinase 1 (CHK1) inhibitors. Among the compounds evaluated, 8n showed potent CHK1 inhibition, with an IC_50_ value of 4.25 ± 0.10 nM, and inhibited the growth of MV-4-11 and Z-138 cells. The authors further showed that compound 8n inhibited CHK1 autophosphorylation in MV-4-11 cells, providing cellular evidence for its target-related activity.^44^

The 2-aminooxazole scaffold has also been investigated in kinase-directed drug discovery. Bae *et al.* reported 2-aminooxazole-based compounds targeting CSF-1R and c-Kit. Their SAR studies showed that structural modifications around the aminooxazole core and its substituents affected kinase inhibitory activity. Selected compounds also inhibited CSF-1-induced ERK phosphorylation in microglial cells, providing evidence of cellular activity.^45^

Representative biologically active 2-aminooxazole derivatives are shown in Fig. 6. Compounds (36) and (37) represent 2-aminooxazole-based compounds investigated in medicinal chemistry, with compound (37) identified as a CSF-1R/c-Kit inhibitor. Compound (38) represents a 2-aminooxazole-containing compound reported for antibacterial and antibiofilm activity. These examples illustrate the diverse biological applications of the 2-aminooxazole scaffold.

**Fig. 6:**
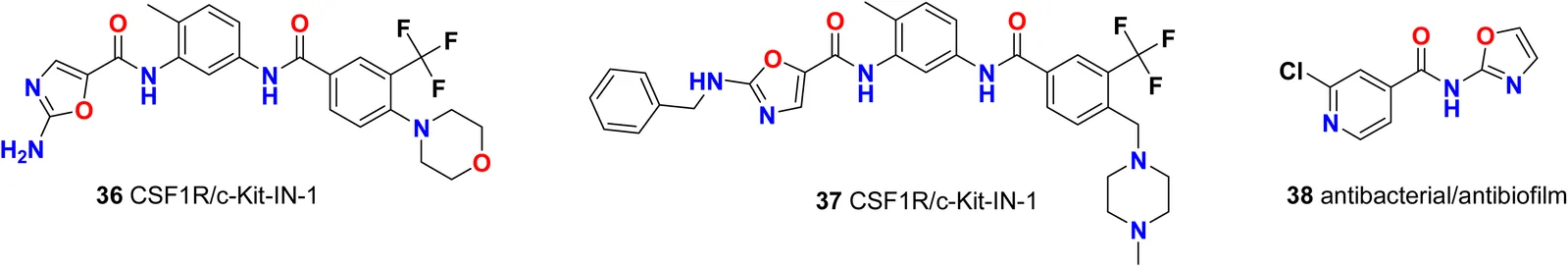
Representative bioactive 2-aminooxazole derivatives and their biological applications.

These examples should not be considered as a direct comparison of potency because the compounds were evaluated against different biological targets and experimental systems. Rather, they demonstrate the versatility of the aminoazole scaffold in biological and medicinal chemistry research. The observed differences in activity also indicate that the biological response depends not only on the aminoazole ring but also on the substituents and overall molecular structure. Therefore, matched structural comparisons are necessary to understand the effects of heteroatom replacement and C4/C5 regioisomerism on biological activity.

### 5.2 Antibacterial and antimycobacterial activities

Modification of the heteroatoms in aminoazole scaffolds can change the physicochemical and biological properties of these derivatives. Juhás *et al.* studied the effect of O-for-S isosteric replacement in related aminoazole compounds and observed changes in antimycobacterial activity.^8^ The effect of the O-for-S replacement is demonstrated by the structures illustrated in Fig. 7. Compound (39), containing the 2-aminothiazole moiety, showed an MIC of ≥500 µg mL^−1^, while the corresponding 2-aminooxazole analogue (40) showed an MIC of 3.125 µg mL^−1^. The second pair, compounds (41) and (42), showed MICs of 3.91 and 7.81 µg mL^−1^, respectively.

**Fig. 7:**
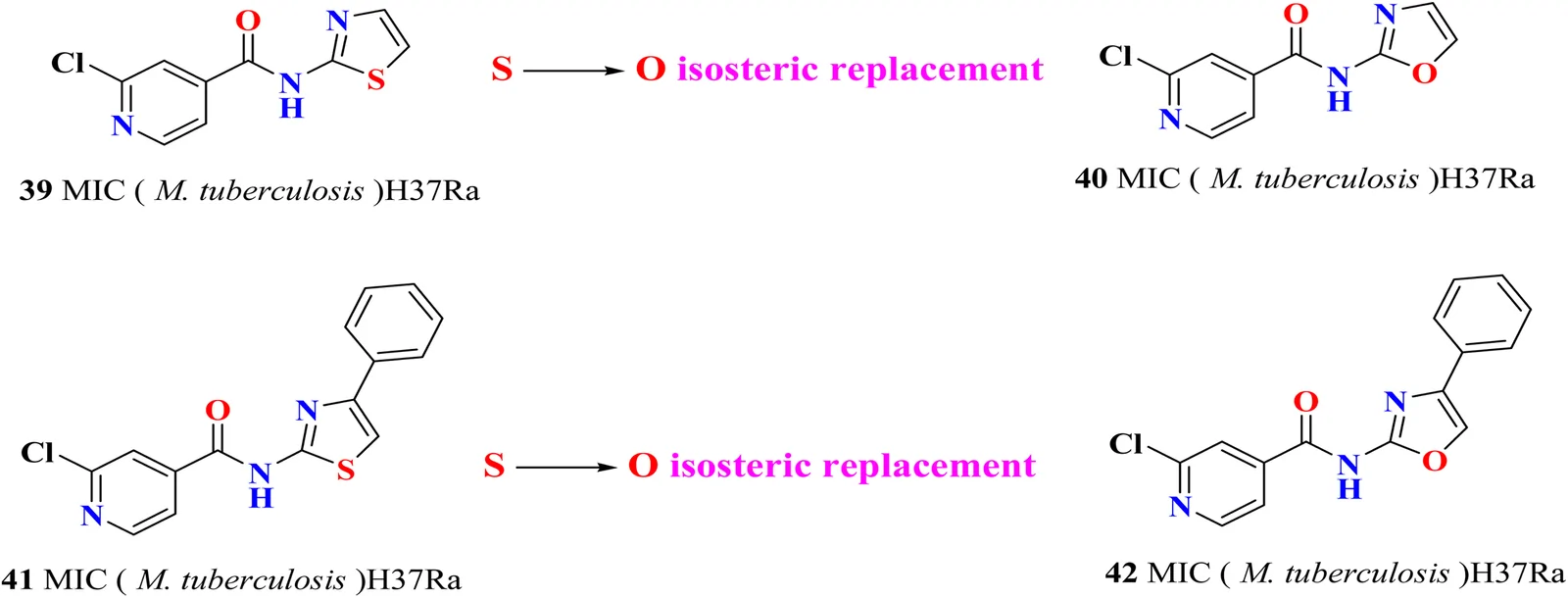
O-for-S isosteric replacement and antimycobacterial activity.

These data show that replacement of sulfur by oxygen can significantly affect biological activity within a defined structural series. However, the effect is compound-dependent, as demonstrated by the different activities observed for compounds (41) and (42). Therefore, these results do not support a general conclusion that 2-aminooxazoles are more active than 2-aminothiazoles, or *vice versa*. The effect of heteroatom replacement is also influenced by substituents, lipophilicity, polarity, and the overall shape and size of the molecule.^8^ The regioisomer–activity relationships of 2-aminothiazoles also remain poorly characterized. Azzali *et al.* showed that modification of the 2-aminooxazole pharmacophore can provide compounds with antitubercular activity against *M. tuberculosis*.^6^ More recently, AB15, a 2-aminooxazole derivative, was reported to show antibacterial and antibiofilm activity against multidrug-resistant *A. baumannii*, with low *in vitro* cytotoxicity. In combination with colistin, synergistic and/or additive effects were observed.^46^ These findings are preliminary and require further investigation before any therapeutic or clinical conclusions can be drawn.

### 5.3 Antifungal activity

2-Aminothiazoles have shown antifungal activity *in vitro*.^47^ In a study of functionally substituted 2-aminothiazoles, molecular docking suggested CYP51 of *Candida albicans* as a potential target, while MurB was proposed in relation to antibacterial activity. The authors appropriately considered these computational results as target hypotheses rather than definitive mechanisms.^47^ The O-for-S modification has the potential to alter lipophilicity, polarity, and biological activity.^8^ The available literature on aminoazoles remains limited in terms of systematic regioisomer–activity relationships. For 2-aminooxazoles, recent biological studies have focused mainly on antitubercular and antibacterial activities.^6,8,46^ Therefore, further experimental antifungal studies, particularly with structurally matched aminoazole derivatives, would be useful.

### 5.4 Antiviral activity

Several aminothiazole derivatives have demonstrated antiviral activity against influenza A. In one study, the position and nature of substituents influenced antiviral activity, and a 4-trifluoromethylphenyl substituent showed significant activity against influenza A/Puerto Rico/8/34 (H1N1).^48^ These results suggest that peripheral substitution can influence antiviral activity and warrants further investigation. Aminothiazoles have also been investigated against enterovirus A71. Compound 12s, with an EC_50_ of 0.27 µM and CC_50_ > 56.2 µM, inhibited viral activity and was shown to interact with the EV-A71 capsid protein, interfering with the interaction between VP1 and the human receptor hSCARB2.^49^ Compared with aminothiazole derivatives, fewer recent studies have examined 2-aminooxazoles for antiviral activity. Further evaluation of appropriately substituted aminooxazoles would therefore be of interest.

### 5.5 Anti-inflammatory and neuroprotective activities

A number of 2-aminothiazole derivatives have been assessed for anti-inflammatory activity. For example, TQS 42 and TQS 43 have been reported to inhibit LPS-induced NO, TNF-α, and IL-6 production in RAW 264.7 cells.^50^ Molecular docking studies with inflammation-related targets were also performed to obtain possible explanations for the observed activity; however, further biochemical and cellular studies are required to confirm these mechanisms. Some 2-aminothiazole derivatives have also shown *in vitro* metal-chelating, neuroprotective, and antioxidant activities, together with acetylcholinesterase inhibition.^51–58^ In the case of aminooxazoles, the reported CSF-1R/c-Kit inhibitors provide a connection with neuroinflammation, as selected compounds inhibited CSF-1-induced ERK phosphorylation in microglial cells.^45^ Thus, the aminoazole framework may provide opportunities for exploring kinase-mediated inflammatory pathways in addition to its established antibacterial applications.

### 5.6 Metabolic and enzyme-inhibitory activities

Some 2-aminothiazole derivatives have shown *in vitro* inhibition of urease, α-glucosidase, and α-amylase, together with antioxidant activity.^52^ Changes in side-chain substitution influenced the observed inhibition, and docking and frontier molecular orbital analyses were used to interpret these results. These findings represent a combination of preliminary *in vitro* experiments and computational analysis. It should be emphasized that enzyme inhibition alone should not be directly linked to therapeutic potential without appropriate cellular, pharmacokinetic, toxicity, and *in vivo* studies. Such evaluation would also require assessment of target selectivity and experimental confirmation of computational hypotheses.^39,52–55^

### 5.7 Structure–activity relationships and regioisomeric effects

The biological activity of aminoazole derivatives depends on the nature of the heterocyclic core as well as the type and position of the substituents attached to it. Electronic and steric effects, together with molecular conformation, can also influence their biological properties.^8,44–49^ In particular, for aminoazoles existing as C4/C5 regioisomers, changing either the substituent or its position on the ring can lead to differences in biological activity. Changes in substitution position can alter the orientation of the molecule and consequently affect its recognition by a biological target.^8^

Because of differences in the spatial arrangement of pharmacophoric groups, C4- and C5-substituted aminoazoles may show different interactions with biological targets. However, it cannot be assumed that one regioisomer is intrinsically more potent than the other unless they are evaluated under the same or closely comparable biological conditions. The activity profile shown in Fig. 8 compares selected 2-aminooxazole and 2-aminothiazole derivatives and illustrates the range of biological responses reported for these scaffolds.

**Fig. 8:**
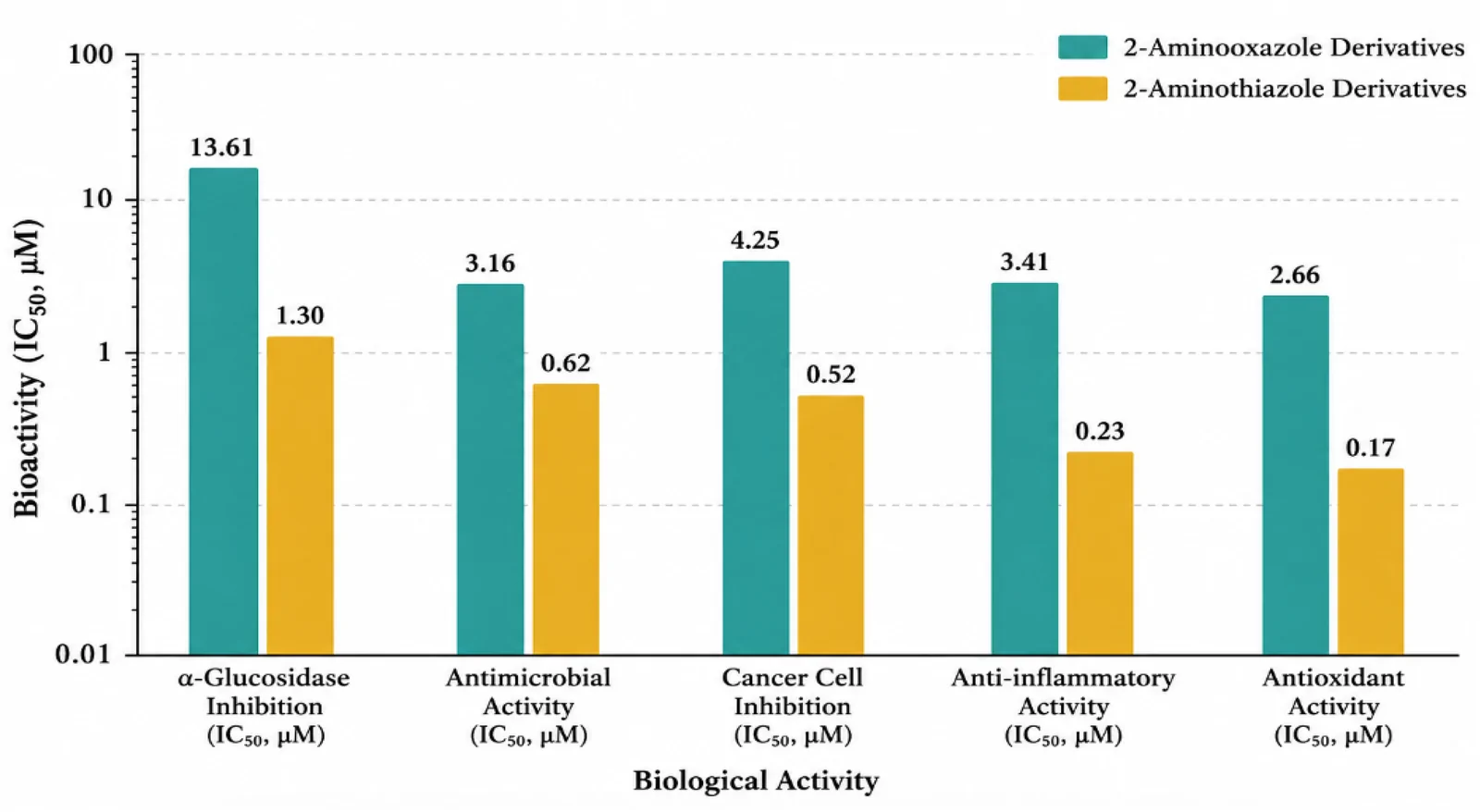
Comparative bioactivity of selected aminoazole derivatives.

Although the values illustrate differences in IC_50_, MIC, or other activity parameters for α-glucosidase inhibition, cancer-cell inhibition, microbial activity, anti-inflammatory activity, and antioxidant effects, these compounds are generally not matched regioisomers. Therefore, before drawing conclusions about the relative biological potency of aminoazole regioisomers, comparative studies using matched compounds should be undertaken while keeping other structural and experimental variables as constant as possible. Such studies would help clarify the relationship between synthetic regioselectivity, molecular structure, and biological recognition.^3,8,19,45^

### 5.8 Preclinical and clinical development

The development status of aminoazole derivatives varies considerably. Alpelisib, a 2-aminothiazole-containing selective PI3Kα inhibitor, provides a notable clinically approved example. It was approved by the FDA in 2019 in combination with fulvestrant for patients with HR-positive, HER2-negative, PIK3CA-mutated advanced or metastatic breast cancer following endocrine therapy.^56,57^

In contrast, AB15 represents a preclinical 2-aminooxazole example investigated against multidrug-resistant *A. baumannii*, while the CSF-1R/c-Kit inhibitors reported by Bae *et al.* represent an early medicinal-chemistry stage of development.^45,46^ Strong *in vitro* activity alone does not establish clinical potential. Further evaluation of pharmacokinetics, toxicology, target engagement, efficacy, and tolerability is required before clinical development can be established.

### 5.9 Critical biological perspective

Several publications have demonstrated a diverse range of biological activities for both 2-aminooxazoles and 2-aminothiazoles. However, the evidence is heterogeneous with respect to the biological target, experimental model, mechanistic verification, and stage of development.^6,44–52,57,58^ Therefore, assignment of general biological activities to either scaffold is not prudent.

The example of O-for-S replacement illustrates how heteroatom substitution can alter the biological activity of a given structure, although the direction and magnitude of this effect are not always predictable.^8^ Similarly, the available evidence for C4- and C5-substituted aminoazoles suggests that regioisomerism can affect molecular orientation and target recognition, but systematic matched studies remain limited.

Computational studies can provide useful clues regarding possible molecular interactions; however, such predictions should be considered as hypotheses that require experimental validation.^8,47,52^ A useful direction for future research is therefore to integrate regioselective synthesis, structural characterization, preparation of matched C4/C5 regioisomers, biological evaluation, and computational analysis. Such an integrated approach could help connect regioselectivity in chemical synthesis with regioisomer-dependent molecular recognition in biological systems. The major biological examples discussed in this section are summarized in Table 4.

**Table 4:** Biological activities and molecular targets of selected aminoazole derivatives

Entry	Activity	Representative scaffold/compound	Target/effect	Evidence level	Key SAR observation	Ref.
1	Anticancer	2-Aminothiazole derivatives	CHK1 inhibition	Biochemical + cellular	Core and peripheral substitution influenced activity	44
2	Kinase/neuroinflammation	2-Aminooxazole derivatives	CSF-1R/c-Kit inhibition; reduced ERK phosphorylation	Biochemical + cellular	N-substitution, amide orientation and pendant-amine topology affected activity	45
3	Antimycobacterial	2-Aminooxazole/2-aminothiazole derivatives	*M. tuberculosis* H37Ra	*In vitro*	O-for-S replacement produced series-dependent changes in activity	8
4	Antibacterial/antibiofilm	AB15	MDR *A. baumannii*; colistin potentiation	*In vitro* + *Galleria*	Structural features of AB15 contributed to antibacterial activity	46
5	Antifungal	2-Aminothiazole derivatives	CYP51/MurB proposed	*In vitro* + computational	Substitution affected activity; targets remain hypotheses	47
6	Antiviral	Aminothiazole derivatives	Influenza A/EV-A71	Cell-based	Peripheral substitution influenced activity	48 and 49
7	Anti-inflammatory	2-Aminothiazole derivatives	NO, TNF-α and IL-6	Cellular + computational	Substitution influenced cellular response	50
8	Neuroprotective	2-Aminothiazole derivatives	AChE inhibition; antioxidant/metal-chelating effects	Enzyme/cellular	Substitution influenced multifunctional activity	51 and 58
9	Enzyme inhibition	2-Aminothiazole derivatives	Urease, α-glucosidase, α-amylase	Enzyme + computational	Structural/electronic modifications influenced inhibition	52
10	Clinical example	Alpelisib	PI3Kα inhibition	Clinically validated	Aminothiazole-containing architecture in kinase-directed therapy	57

## 6 Integrated mechanistic, computational and structure–activity framework

The combined consideration of regioselectivity, molecular architecture, biological activity, and sustainability has been demonstrated throughout the previous sections. The formation of 2-aminooxazoles and 2-aminothiazoles varies mostly due to competing cyclization pathways, type of substituents, and the nature of catalyst and substrate.^27,31,59–65^ This section integrates mechanistic and biological considerations to explain the biological activities and potential responses of the regioisomers formed during synthetic processes.

### 6.1 Mechanisms of regioselectivity

Various pathways to the same end product are possible in synthetic transformations. For unsymmetrical substrates, activation at different sites can lead to regioisomeric products. Regioselectivity is influenced by a combination of substituent effects, electronic properties, steric factors, conformational preferences, and reaction conditions. The relative stability of intermediates and transition states can therefore influence the distribution of regioisomeric products. These factors are particularly relevant to aminoazole synthesis, where alternative cyclization or rearrangement pathways may lead to different regioisomeric outcomes.^39,60^ Regioselectivity can also be examined through computational studies. DFT calculations allow the relative energies of competing intermediates and transition states to be compared. Differences in the corresponding activation barriers may help explain why one regioisomer forms preferentially. The calculated energy profile, however, can change with the computational method, solvent model, catalyst representation, and reaction conditions. Computational results should therefore be treated as mechanistic support rather than as conclusive proof. Experimental identification and analysis of the products are still needed to confirm the predicted pathway.^60,61^ Electronic-structure calculations can also be useful when comparing the biological behaviour of related aminoazole derivatives. HOMO–LUMO energy gap (Δ*E*), chemical hardness (*η*), dipole moment, and frontier molecular orbitals provide information about their electronic characteristics. In the series examined, changes in these parameters were associated with differences in biological activity. Such relationships should be interpreted within the specific compound series, however, and should not be extended to all 2-aminooxazole and 2-aminothiazole derivatives. The comparative HOMO–LUMO characteristics, chemical hardness, dipole moment, and their relationship with biological activity are presented in Fig. 9.^61^ The chemical hardness can be expressed approximately as *η* ≈ Δ*E*/2, where Δ*E* represents the HOMO–LUMO energy gap. A larger gap generally indicates greater resistance to electronic perturbation, whereas a smaller gap indicates greater electronic flexibility. Nevertheless, electronic descriptors alone cannot establish a direct relationship between regioselectivity and biological activity. They should therefore be considered as supporting parameters for mechanistic interpretation and structure–activity relationship (SAR) analysis. The correlations shown in Fig. 9 should consequently be understood as relationships observed within the analysed compounds rather than universal trends.^61–69^

**Fig. 9:**
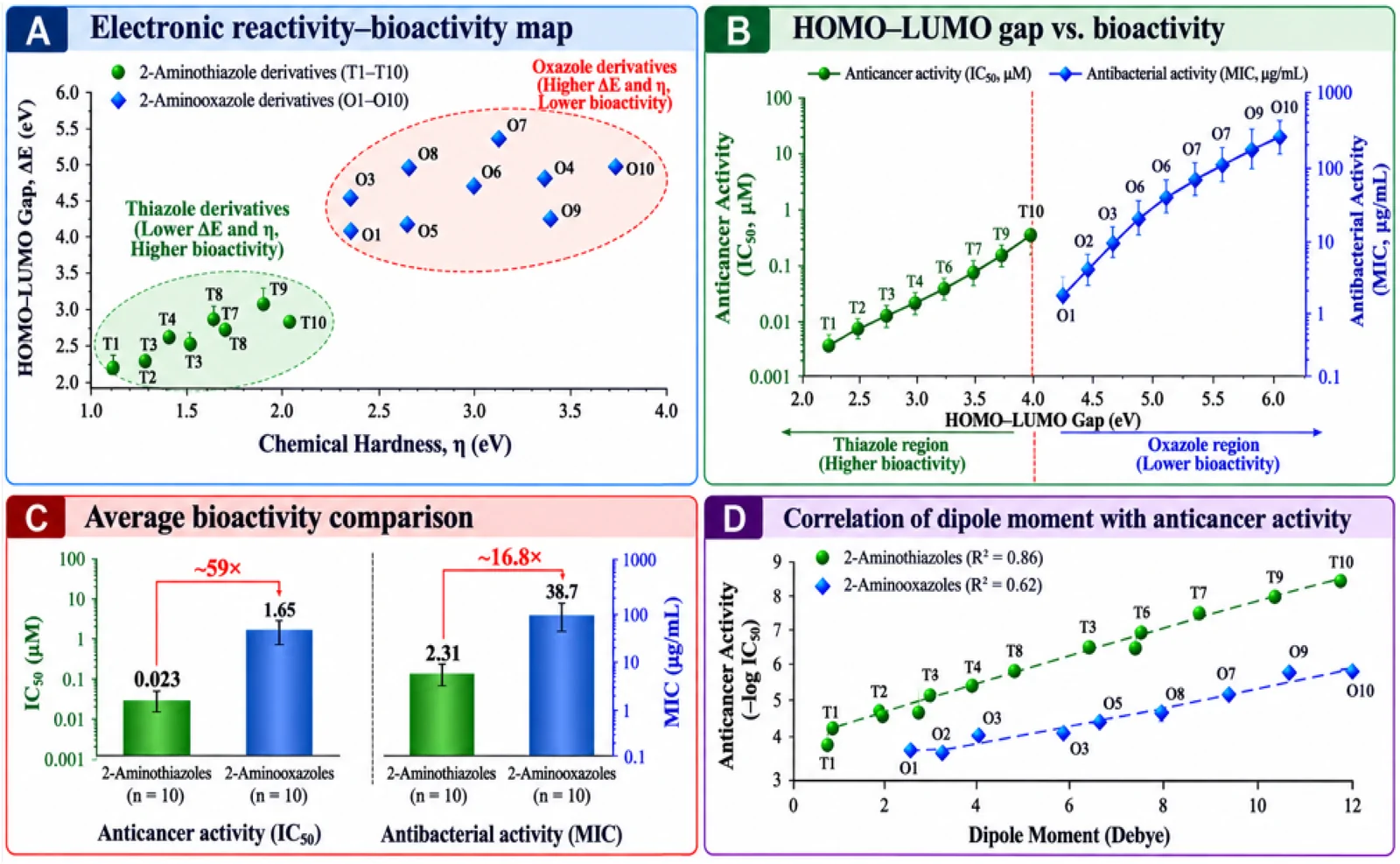
Comparison of electronic properties and biological activities of selected 2-aminothiazole and 2-aminooxazole derivatives: (A) chemical hardness and HOMO–LUMO gap; (B) relationship between the HOMO–LUMO gap and anticancer and antibacterial activities; (C) average anticancer and antibacterial activities of both compound classes; and (D) relationship between dipole moment and anticancer activity. These trends are based on the selected dataset and may not apply to all compounds.

### 6.2 Computational structure–activity and AI-assisted prediction

Molecular modelling incorporates additional data relevant to biological activity and regioselectivity. Docking studies analyze the possible interactions between a ligand and a target, including hydrogen bonding, π-interaction, and hydrophobic contacts, and other non-covalent interactions. Examining different regioisomers can be used to develop reasonable speculations concerning the possible binding orientation and interactions with the biological target. Predicted interactions and bonding, however, should be corroborated by experimental data. The majority of the studies identified in the present review use a combination of different computational methods. While DFT and molecular docking were the most frequently identified approaches, the use of molecular dynamics, QSAR/descriptors, and AI/ML methods for simulation and optimization were also evident in the literature surveyed [Fig. 10].^16,43^ Because the same study may encompass multiple methods, the values presented in the figure, reflect cases rather than exclusively separate studies.

**Fig. 10:**
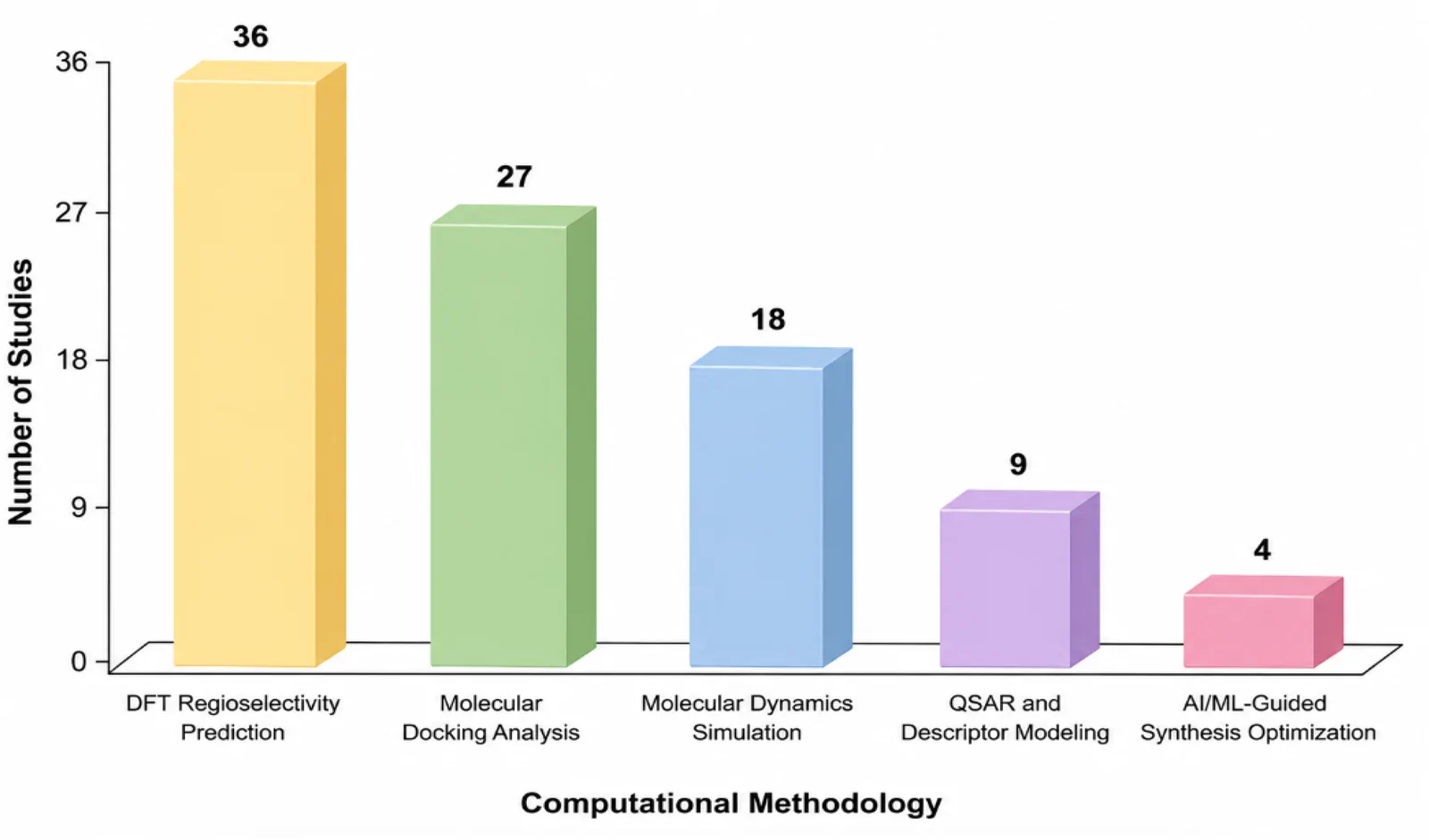
Distribution of computational approaches identified in the studies surveyed. Categories are not necessarily mutually exclusive, as individual studies may employ multiple approaches.

DFT calculations can be used to compare the energies of competing reaction pathways. Molecular docking can help to understand the possible interactions between a compound and a target. Further information about the resulting complexes can be gained through molecular dynamics simulations. QSAR and other molecular-descriptor approaches can help in understanding the relation between molecular structure and biological response. Recent studies help to illustrate the effect of molecular properties and biological activity, as well as the factors that determine synthetic selectivity.^59,69^ These studies reflect the current trend towards integrating molecular properties, synthetic selectivity, and biological activity, even if these approaches provide evidence in support of the activity and/or binding rather than providing proof.^59,69^ Other trends in the field are the use of AI/ML to discover patterns in large data sets and help synthesize and rank substrates and products as well as assist in designing optimal reactions and conditions.^55,65–74^

A significant methodological example is provided by Guan *et al.*, who integrated machine learning and quantum chemistry through a machine-learning workflow whereby DFT reaction modeling was invoked for predictions with lower machine-learning confidence. The approach was demonstrated for SNAr regioselectivity, illustrating the potential integration of machine learning and quantum chemistry in chemistry.^43^ As this work is focused on SNAr chemistry rather than aminoazole synthesis, this serves more as a methodological example than as evidence directly relating to the regioselectivity in the aminoazole synthesis. For aminoazole chemistry, such a framework could be developed by integrating regioisomer distributions determined through experiments with molecular, electronic, steric, and other reaction-condition descriptors. Development of such models would require a dataset representing a diverse range of regioisomers that are supported through reliable experiments. Hence, the emphasis should be on experimentally verifying AI/ML generated predictions, and not on purely relying on predictions for new chemistry.^43,59,69^

### 6.3 Energy profiles and catalytic effects

Catalysts can provide an alternative reaction pathway with a lower activation free energy by stabilizing the transition state or reactive intermediate. Such a reduction in the activation barrier can facilitate the reaction under the investigated conditions. The conceptual difference between catalysed and uncatalysed pathways is illustrated in Fig. 11.^39^ However, a reduction in activation energy alone does not establish regioselectivity. When two or more competing pathways are possible, regioselectivity requires comparison of the corresponding activation barriers and the experimentally observed product distribution. Thus, a catalyst may accelerate a reaction without necessarily determining which regioisomer is preferentially formed. A mechanistic interpretation of catalyst-dependent regioselectivity should therefore consider the relative energy profiles of the competing pathways together with experimental product characterization.^60,61^ This distinction is important when evaluating green and catalytic aminoazole syntheses. Improved reaction rate, shorter reaction time, or lower energy consumption may contribute to process efficiency, but these parameters should not automatically be interpreted as evidence for improved regioselectivity. Similarly, high isolated yield does not necessarily establish selective formation of a particular regioisomer unless the product distribution has been experimentally determined.

**Fig. 11:**
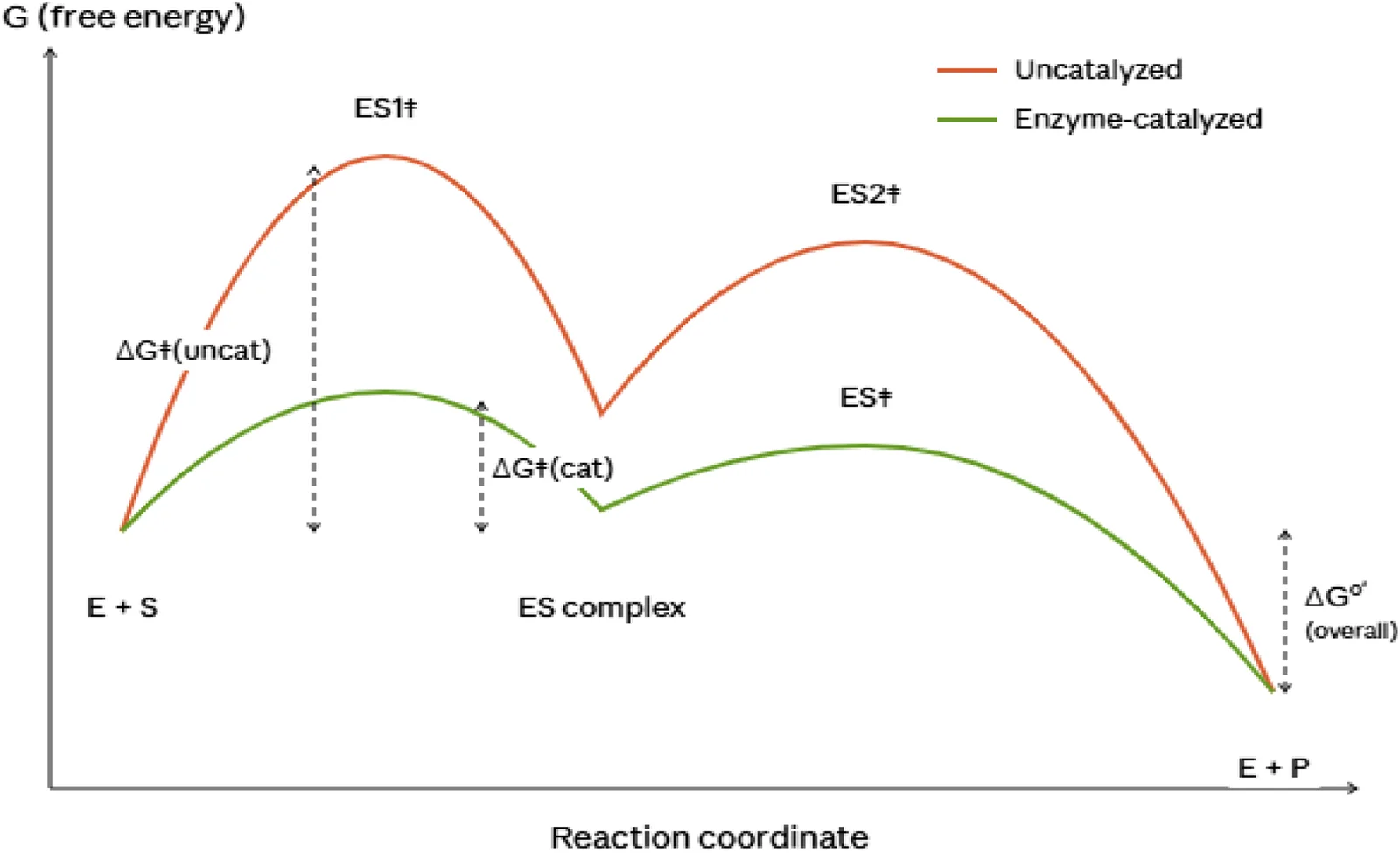
Schematic energy profiles showing catalytic reduction of the activation free energy relative to the uncatalysed pathway; the profile alone does not establish regioselectivity.

The integration of mechanistic calculations with experimental product analysis is therefore particularly valuable. Computational energy differences can provide a possible explanation for the observed selectivity, whereas spectroscopic, chromatographic, crystallographic, or other structural analyses establish the identity and distribution of the products. This combined approach provides a stronger basis for assigning regioselectivity than either computational prediction or isolated yield alone.

### 6.4 Translational, catalyst and process constraints

There are several constraints when transitioning a synthesis from the lab-scale to commercial-scale, and regioselectivity is only one of these constraints. Mass transfer and heat transfer can affect the overall performance of a chemical reaction. This means that parameters such as the reaction volume, mixing, and residence time can have an impact on the performance of a large-scale chemical reaction, and controlling these parameters can be difficult. Some of these challenges can be managed better when using Process Intensification (PI) techniques, but these techniques must be evaluated for each individual reaction system to be validated.^39^ The environmental performance of a catalytic or regioselective process needs to be assessed beyond isolated yields. Factors such as solvent use, reagent excess, catalyst loading, energy use and waste can affect the overall performance and sustainability of a process. Regioselectivity and reaction yield should be assessed in the context of the overall process, including the burden placed on the process. Process intensification is especially critical when a regioselective aminoazole synthesis is attempted at a larger scale. Continuous-flow processes allow better control of reaction conditions and can help improve heat and mass transfer. This is especially useful when addressing challenges related to larger-scale synthesis of aminoazoles.

The dependence of reaction chemistry and engineering parameters leads to experimental determination of the relations for each transformation.^39^ One such study integrated the synthesis of substituted 2-aminothiazoles and used DFT calculations, molecular docking and ADME/Tox. Serdaroğlu, *et al.*, reported the electronic and molecular properties of the products using DFT, and determined their potential biological activities with molecular docking studies and evaluated their drug likeliness using ADME/Tox. They also presented a general approach for further studies with examples from literature. Predicted reaction pathways, binding modes of ligands to proteins, activity and selectivity profiles need experimental confirmation, and until they do, they should not be the basis for drawing mechanistic or pharmacological conclusions.^42^ The improvement of the synthetic routes to aminoazoles is considered taking into account regioselectivity, synthesis efficiency, biological activity and practical aspects related to the synthetic process. An analysis considering these factors together provide a more complete information for the utility of a synthetic route rather than taking into account any parameter alone, including the yield.

### 6.5 Integrated assessment, green metrics and research gaps

It is not always straightforward to compare the aminoazole syntheses that are reported in the literature, since different reaction conditions are employed. Many factors can affect the efficiency of a synthetic method such as the scale of the reaction, the solvent, the loading of the catalyst, the duration of the reaction, the work-up, and the purification. The isolated yield, therefore, does not capture the full picture on the sustainability of a particular synthetic method. Other metrics such as the atom economy, process mass intensity (PMI), E-factor, the consumption of energy and the type and quantity of the solvent or catalysts are also important.^38,62^ The quantity of waste generated during work-up and purification is particularly important, and can be significantly more than that produced during the synthesis step. Life cycle assessment (LCA) can give an idea about the impact of a certain synthesis on the environment and consider all stages of the process.^38,62–64^ A reaction with a high yield can be less sustainable than a comparable alternative.

These considerations are interconnected in aminoazole synthesis. For example, in some cases a reaction may give an excellent yield; however, it predominantly forms a regioisomer which is of less or no biological activity. Moreover, high selectivity may require the employment of high energy and/or expensive reagents as well as hazardous or inconvenient reaction conditions which may lead to extra labor for purification. Computational studies may predict some of these differences by looking at reaction pathways, electronic features and biological target interactions. In some cases, their predictions can be verified. Similar considerations apply to green chemistry metrics: their value depends on the quality and completeness of the process data used for calculation rather than on reaction yield.

The literature also points to a lack of studies that consider these aspects in an integrated manner. Several methods that have been reported focus on obtaining the desired aminoazole, and, thus, the corresponding comparative biological studies are lacking. In general, detailed studies of regioselectivity and biological activity are lacking. Given the lack of this fundamental knowledge, it is not always clear how regioselectivity of the syntheses affects biological activity and what kinds of additional developments are necessary.

## 7 Future perspectives

Consideration should be given to regioselectivity during the synthesis of aminoazoles and what types of differences in biological activity would be of interest. This could drive improvements in 2-aminooxazoles and 2-aminoazoles and how they are evaluated. For aminoazoles where regioselectivity leads to different biological activity and selectivity, computational studies can potentially help prioritize targets, but experimental studies remain essential. Other aspects such as the use of solvents, the need for energy, catalysts, purification and scale-up should be addressed, in addition to the selectivity of the regioselective reactions.^39,54–56,64–70^

### 7.1 Regioselective and bioactive design using AI and DFT

There are many factors that can affect the outcome of an aminoazole cyclization. These include differences in the structure of the substrate, type of catalyst, type and polarity of the solvent, temperature, and other conditions of the reaction. The relative energy of the various pathways can impact the outcome of the reaction.^68–85^ DFT calculations can be utilized to predict the energies of the pathways by calculating the structures of the intermediates and transition states along the various pathways. This can provide insight into pathway preferences and regioselectivity. If the calculations are supported by experimental data, they can be used to explain the preference of one regioisomer over the other.^59,69^

Methods based on AI and ML are also being developed to predict reactions, design molecules, and analyze structure-activity relationships.^55,65,67–70,74,85–96^ DFT-based descriptors may help identify characteristics in molecules responsible for regioselectivity and biological activity when integrated with experimental data on reactions and biology.^55,65,67–70^ More recently, computational studies showed how the consideration of synthesis, molecular, and biological properties allowed researchers to define criteria for bioactive compound design, and thus design more rationally active molecules.^59,69^ Such models, however, should be validated by new experimental data.

Considering regioisomers in pairs is of utmost importance. When regioisomers differ only in position of substitution, differences in their biological activities can be more confidently associated with regioisomerism.^68,71,80,85,97–107^ It should also be mentioned that there are alternative methods, beyond target-based assays, available to determine biological activity, for example, cellular assays and ADME evaluations.

Fig. 12 presents a possible workflow linking computational analysis with synthesis and biological evaluation. DFT calculations can be used to examine competing reaction pathways, while AI/ML methods may help in selecting substrates, predicting regioselectivity, or prioritizing compounds for synthesis.^55,59,65–69,83,85^ The synthesized regioisomers can then be characterized and compared in biological assays and SAR studies, followed where appropriate by ADME evaluation.^65,71,76,80,98–107^ The synthetic procedure can be examined separately using green metrics, process-intensification methods, and scale-up studies.^60,61,66,77,89^ Results obtained experimentally can then be used to improve the next round of computational prediction and synthesis. This framework is intended as a research strategy rather than as a validated predictive model. Experimental evidence is still required to confirm conclusions about regioselectivity, biological activity, and environmental performance.^55,67,71,76,80,85^

**Fig. 12:**
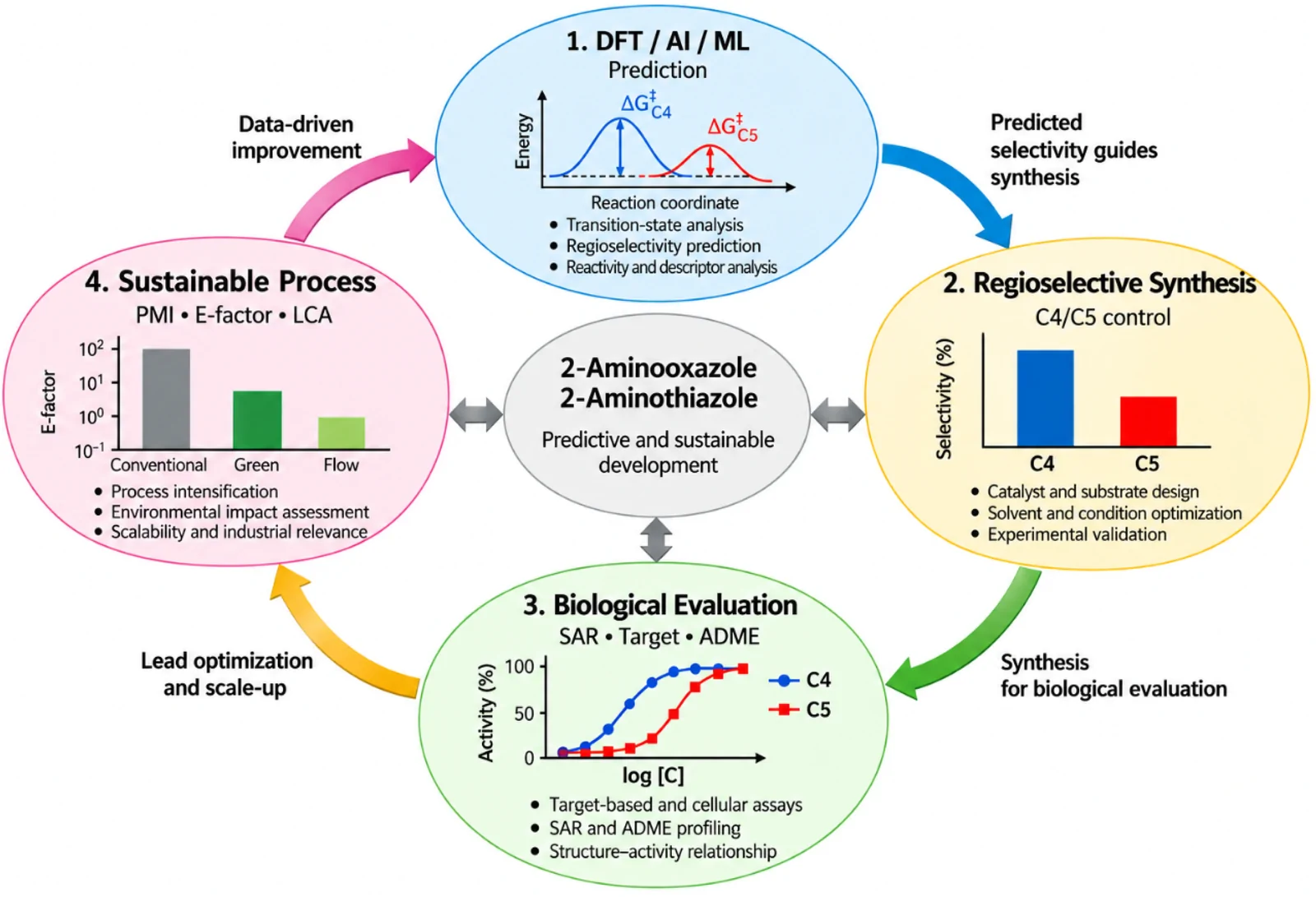
Integrated predictive and sustainable framework for aminoazole development.

### 7.2 Sustainable process development and translational scale-up

Yield is just one factor. Reagent and catalyst consumption, the energy and water/solvent requirements, and process waste all impact the overall sustainability of a synthesis. The control of a reaction during scale-up relies heavily on process analytical technology (PAT).^39,54,65–67,88,89^ PAT can be used in conjunction with process simulation to better understand the effect of processing and reactor/plant design on the overall process.

There is a need to look at processes beyond just isolated yields, and this is especially true for synthetic methods involving nanocatalysis, multicomponent and domino syntheses, aqueous/solvent-minimized and microwave and ultrasound syntheses. There are many process-related challenges to synthesizing multicomponent and domino syntheses at scale. These include catalyst recovery, reagent/product separations, and purification. There are process-related challenges to synthesizing aqueous and solvent-minimized reactions at scale. These include energy and solvent requirements. Not all solvents or methods for solvent removal are equally effective or sustainable. Considering conversion, regioselectivity, and selectivity and the quality of the final product provides a more complete picture of a synthesis. Assessing the process includes determination of atom economy, PMI, *E*-factor, LCA, catalyst loadings and recovery, solvents and energy consumption, and other input and output components of a synthesis.^60,61,66,77,89^

### 7.3 Integrated research gaps and future opportunities

While progress is discussed above, several areas of aminoazole chemistry remain unconnected to allow for reliable prediction. These include the systematic comparison of C4/C5 regioisomers, and the impact of changing the ring heteroatom is not understood across different chemical series. The same is true in biological studies where regioisomer pairs are not always available, and in the synthesis, computation, biology and process domains where the information is often published in isolation. Integration of these would allow a more reliable prediction of molecular structures and regioselective synthesis, and their relation to biological activity and process performance.^60,61,66,77,88,89^

The main research directions that have emerged from this review are summarized in Table 5. The most important of these are considered to be the integration of ‘divide-and-conquer’ synergistic approaches where mechanism-based biological investigations are coupled to sustainable synthesis and multi-objective scaling and optimization, as well as predictive artificial intelligence/machine learning models.

**Table 5:** Research gaps, current limitations, and future opportunities in aminoazole chemistry

Entry	Research area	Current limitation	Future opportunity	Ref.
1	C4/C5 regioselectivity	Selectivity remains substrate- and condition-dependent	DFT- and mechanism-guided prediction with experimental validation	67, 68, 83, and 85
2	AI/ML modelling	Limited validated and chemically diverse datasets	Prospective validation using substrate, reaction-condition, and regioisomer-distribution data	55, 65, 67, 69, 70, 74, and 85
3	Regioisomeric SAR	Limited systematic matched C4/C5 comparisons	Matched-pair studies to distinguish positional effects from other structural changes	71, 76, 80, and 98–107
4	Biological translation	Uneven mechanistic, ADME, and *in vivo* evidence	Sequential target, cellular, selectivity, ADME, and *in vivo* validation	71, 76, 80, and 98–107
5	Sustainable synthesis	Inconsistent reporting of process-level sustainability	Standardized PMI, E-factor, solvent, energy, catalyst-recovery, and LCA assessment	60, 61, 65, 66, 77, and 89
6	Scale-up	Limited evidence for long-term process robustness	Continuous-flow and PAT-assisted development with experimental scale-up	39, 67, 88, 89, and 92
7	Multi-objective optimization	Chemical and environmental parameters often evaluated separately	AI-assisted simultaneous optimization of selectivity, efficiency, and sustainability	55, 65, 66, 77, 84, and 85
8	Data integration	Reaction, computational, and biological datasets remain fragmented	Integrated mechanistic–synthetic–biological datasets for predictive modelling	67, 69, 71, 73–75, and 79

## 8 Results and discussion

This review examines literature relating to the synthesis of 2-aminoazoles. It shows that a variety of techniques have been utilized for the synthesis of 2-aminoazoles including those that employ established cyclization methods, catalytic methods, solvent-minimized methods, microwave-assisted methods, ultrasound-assisted methods, and/or continuous-flow methods. Use of the above methods, in many cases, has increased the rate of reaction and simplified the synthesis of 2-aminoazoles. However, an improved isolated yield and/or a shorter reaction time does not always indicate that a synthesis has been improved. The behavior of the substrate, catalyst, and solvent, in combination with the reaction conditions can affect the selectivity of the reaction. For this reason, the synthetic chemist should consider the overall reaction in addition to yield and reaction time.

Regioselectivity, for example, may lead to the formation of multiple cyclization products resulting in a lack of control over the desired outcome. The use of a range of electronic and steric effects, in combination with the type of catalyst, may influence the pathway that is preferentially followed. Formation of unwanted regioisomeric byproducts may necessitate increased chromatographic purification. In this way, poor regioselectivity may lead to increased reaction times and/or solvent consumption and may adversely affect the overall synthetic efficiency.

Various recent methods have attempted to resolve the above-mentioned issues by using catalytic activation, solvent-free or solvent-min conditions, microwave or ultrasound irradiation, recyclable catalysts and continuous-flow processes. While these methods are interesting, the reaction medium or the way the activation is performed in itself should not be the basis to analyze the sustainability of the method. One should also take into account the volume of solvents used, excess of reagents, amount of catalyst, energy consumption, ease of work-up, purification, and recovery of the catalyst and the scale-up of the process. When data are available, assessment of the process based on atom economy and other parameters (*e.g.* process mass intensity (PMI) and E-factor) is of greater interest. Solvent and energy consumption should be included as well. Life cycle assessment may also be of interest.

The biological literature also illustrates the same type of problems when various aminoazole derivatives are assessed. It has been shown that several aminoazole derivatives exhibited significant anticancer, antibacterial, antimycobacterial, antiviral, and anti-inflammatory activities. Some derivatives showed neuroprotective and enzyme-inhibitory activities. These findings are interesting, but there are great variations in the derivatives assessed, target of the assay, the protocol of the assay and the range of concentration of the derivatives, and the level of validation. Thus, activities of aminoazole derivatives reported in unrelated series should not be directly compared.

The comparison becomes more useful when looking at structurally equivalent compounds. For instance, in the case where both the C4- and C5-regioisomers can be prepared and tested in the same assays, the effects of position of the substituent *versus* other structural changes can be determined. Related studies are not very common. The position of the substituent can determine the shape and/or the electronic distribution of the molecule and can influence the physicochemical properties of the molecule. These changes can lead to a change in the way a molecule interacts with its biological target, and consequently, to a change in its biological activity.

Computational methods can be quite useful in this regard. For example, the comparison of competing pathways in a reaction and their associated transition states can be analyzed by DFT methods. Potential binding modes can be evaluated by molecular docking studies. QSAR analyses and molecular descriptors can be used to explain observed activity. However, reliance on these approaches should be taken with caution as their reliability depends on the quality of the structures and measurements. Therefore, a low docking score or a positive correlation in a dataset should not be interpreted as biological activity.

Computational methods have recently been developed to predict the outcome of reactions and to assist in the selection of reaction substrates, the determination of regioselectivity, and in the evaluation of structure-activity relationships. The main limiting factor for the use of computational methods will be the availability of quality and consistent data. For aminoazole methodology, in addition to substrate structures, data sets that include reaction conditions, regioisomer ratios, biological data, process data, and other relevant information would be very useful. Currently the data required for the development of model systems is often reported in an insufficient or inconsistent format and across multiple publications. Therefore, rather than advancing aminoazole chemistry by developing new methods, a better goal would be to describe the relationships between the selectivity of the reactions, molecular structures and biological activity.

Synthetic methods are re-evaluated for regioselectivity, substrate scope, purification requirements, and the materials, energy, solvents, and catalysts required. Biological claims also become more compelling when related regioisomers are compared under the same or similar conditions. Initial activity should be corroborated with either mechanistic or biologic validation.

Taking the entire picture into account helps to determine when additional research is warranted. The regioisomer formed is determined largely by reaction conditions. The substitution pattern can alter other molecular properties and the biological response. The synthetic pathway can impact how easily a compound can be produced on a large scale. While considering all of these factors, the “art” of synthesis can be applied to determine which aminoazole structures have the most potential and which methods are attractive only on a reaction-scale basis. Such a perspective is most applicable to syntheses of compounds that are of particular interest from a biological or process perspective.

## 9 Conclusion

2-Aminooxazoles and 2-aminothiazoles have significant synthetic and medicinal value. Multiple methods have been developed beyond the traditional cyclization methods. For example, the development of continuous-flow technology has provided further methods, including one-pot, microwave-assisted and ultrasound-assisted methods. When comparing these methods, many factors other than yield and time should be considered. Some of these include substrate scope, purification requirements, regioselectivity, solvent and energy requirements, and the potential for scale-up.

Regioselectivity is a significant issue for systems that result in the formation of C4- and C5-substituted products. In many cases the two regioisomers can display significantly different chemical and biological behavior. This can be determined by using synthesized regioisomers in a pair-wise comparison. Due to this, S–A relationships may be better supported by systematic C4/C5 regiospecific syntheses performed in congruence with selected biological screening.

The same can be said when evaluating green chemistry principles. While the use of certain methodologies such as microwave irradiation, ultrasound, flow processing, and other catalytic methods may improve the practicality of a synthesis, they do not inherently ensure that a synthetic method is greener. It is essential that the consideration of all aspects of a synthesis be evaluated together including energy, solvent and reagent consumption, waste generation, purification requirements, scale-up, and catalyst recovery. Where experimental data are available, metrics such as PMI and E-factor can be utilized.

Computational methods provide another perspective to aminoazole research. As mentioned before, DFT can help analyze competing reaction pathways, and docking, QSAR, and AI/ML can help analyze molecular interactions and structure-activity relationship. Their conclusions can be drawn more confidently when their predictions are verified by experimental observations and when the datasets upon which their conclusions are based are sufficiently large and diverse.

In the future, it will be beneficial if several of these aspects (selective synthesis, structural characterization, biological assessment, computation, and process) are integrated within the same research program. Evidence suggesting C4/C5 regioisomer selectivity, addressing competing cyclization pathways, building and experimentally validating computational models, and other process-related data would help address the gaps described in this review.

## Author contributions

The study was conceived and designed by all authors. The material preparation, data collection, and analysis were performed by Rohini Dattatraya Patare and Dr Kailas Khandu Sanap. The first draft of the manuscript was prepared by Pankaj Popatrao Yenare and subsequently improved by contributions from all authors. All authors read and approved the final manuscript. Correspondence related to this article should be addressed to Dr Kailas Khandu Sanap.

## Conflicts of interest

The authors have no competing interests to declare.

## Data Availability

No new experimental or computational data were generated in this review. All information discussed is available from the cited literature.
